# Healthcare-seeking behavior for respiratory illnesses in Kenya: implications for burden of disease estimation

**DOI:** 10.1186/s12889-023-15252-3

**Published:** 2023-02-16

**Authors:** Gideon O. Emukule, Eric Osoro, Bryan O. Nyawanda, Isaac Ngere, Daniel Macharia, Godfrey Bigogo, Nancy A. Otieno, Sandra S. Chaves, M. Kariuki Njenga, Marc-Alain Widdowson

**Affiliations:** 1 US Centers for Disease Control and Prevention - Kenya Country Office, KEMRI Headquarters, Mbagathi Rd, Off Mbagathi Way, Village Market, P.O Box 606, Nairobi, 00621 Kenya; 2 Washington State University Global Health, Nairobi, Kenya; 3 Kenya Medical Research Institute, Kisumu, Kenya; 4 Influenza Division, National Center for Immunization and Respiratory Diseases, Centers for Disease Control and Prevention, Atlanta, GA USA; 5 Institute of Tropical Medicine, Antwerp, Belgium

**Keywords:** Behavior, Burden, Healthcare seeking, Healthcare utilization, Kakamega, Marsabit, Nakuru, Pneumonia, Respiratory illness, Siaya, Kenya

## Abstract

**Background:**

Understanding healthcare-seeking patterns for respiratory illness can help improve estimation of disease burden and target public health interventions to control acute respiratory disease in Kenya.

**Methods:**

We conducted a cross-sectional survey to determine healthcare utilization patterns for acute respiratory illness (ARI) and severe pneumonia in four diverse counties representing urban, peri-urban, rural mixed farmers, and rural pastoralist communities in Kenya using a two-stage (sub-locations then households) cluster sampling procedure. Healthcare seeking behavior for ARI episodes in the last 14 days, and severe pneumonia in the last 12 months was evaluated. Severe pneumonia was defined as reported cough and difficulty breathing for > 2 days and report of hospitalization or recommendation for hospitalization, or a danger sign (unable to breastfeed/drink, vomiting everything, convulsions, unconscious) for children < 5 years, or report of inability to perform routine chores.

**Results:**

From August through September 2018, we interviewed 28,072 individuals from 5,407 households. Of those surveyed, 9.2% (95% Confidence Interval [CI] 7.9–10.7) reported an episode of ARI, and 4.2% (95% CI 3.8–4.6) reported an episode of severe pneumonia. Of the reported ARI cases, 40.0% (95% CI 36.8–43.3) sought care at a health facility. Of the74.2% (95% CI 70.2–77.9) who reported severe pneumonia and visited a medical health facility, 28.9% (95% CI 25.6–32.6) were hospitalized and 7.0% (95% CI 5.4–9.1) were referred by a clinician to the hospital but not hospitalized. 21% (95% CI 18.2–23.6) of self-reported severe pneumonias were hospitalized. Children aged < 5 years and persons in households with a higher socio-economic status were more likely to seek care for respiratory illness at a health facility.

**Conclusion:**

Our findings suggest that hospital-based surveillance captures less than one quarter of severe pneumonia in the community. Multipliers from community household surveys can account for underutilization of healthcare resources and under-ascertainment of severe pneumonia at hospitals.

**Supplementary Information:**

The online version contains supplementary material available at 10.1186/s12889-023-15252-3.

## Background

Acute respiratory infections are a major contributor to morbidity and mortality globally [1, 2]. Although most cases of acute respiratory infections do not require medical attention, many people may need healthcare, and some will develop severe disease requiring hospitalization or ventilation assistance. Early treatment will reduce the likelihood of the most severe disease outcomes including death, but in low- and middle-income countries (LMIC) settings, people do not seek care for acute respiratory illness (ARI), even when their clinical presentation is severe [3–5]. Use of public or private healthcare facilities depends on socio-economic factors, cultural beliefs and practices, distance, availability, affordability and quality of healthcare [3, 4, 6–8]. In Kenya, lack of access to healthcare facilities due to various factors such as distance, cost of travel and user fees may lead persons to seek alternative options for healthcare, like pharmacies and traditional healers [8–10]. Inaccurate or unavailable data on local disease burden data of persons with severe illness who do not seek care at healthcare facilities challenges prioritization of control measures, especially in LMICs and may also lead to substantial underestimation of the impact of interventions, such as vaccination.

Healthcare utilization surveys (HUS), have been conducted in several resource-limited countries to determine the healthcare utilization practices for specific diseases in defined populations [3–5, 11]. In addition, data from HUS can be used to improve estimation of disease incidence and burden estimation, to understand barriers to seeking care and to identify alternative healthcare systems that should be included in disease surveillance systems. In Kenya, HUS data are limited to certain well defined specific populations such as Siaya County in rural western Kenya [3, 8] and informal settlements in the capital city of Nairobi and on the coast [8, 12]. The Kenya Demographic and Health Surveys (KDHS), which are conducted every 4–5 years provide estimates of healthcare utilization data for mild diarrheal and acute respiratory illness (ARI) among children < 5 years; however, they do not capture severe illness and are not sufficiently powered for regional estimates or to estimate illness in other age groups [13].

Between August and September 2018, we conducted a community survey of healthcare utilization for respiratory illness among residents of four diverse counties in Kenya. Here, we estimated the proportion of respiratory illness episodes in these communities that sought care at a clinic or hospital (including hospitalization) and assessed factors associated with seeking healthcare at a health facility for respiratory illness.

## Methods

### Study sites

**Fig. 1:**
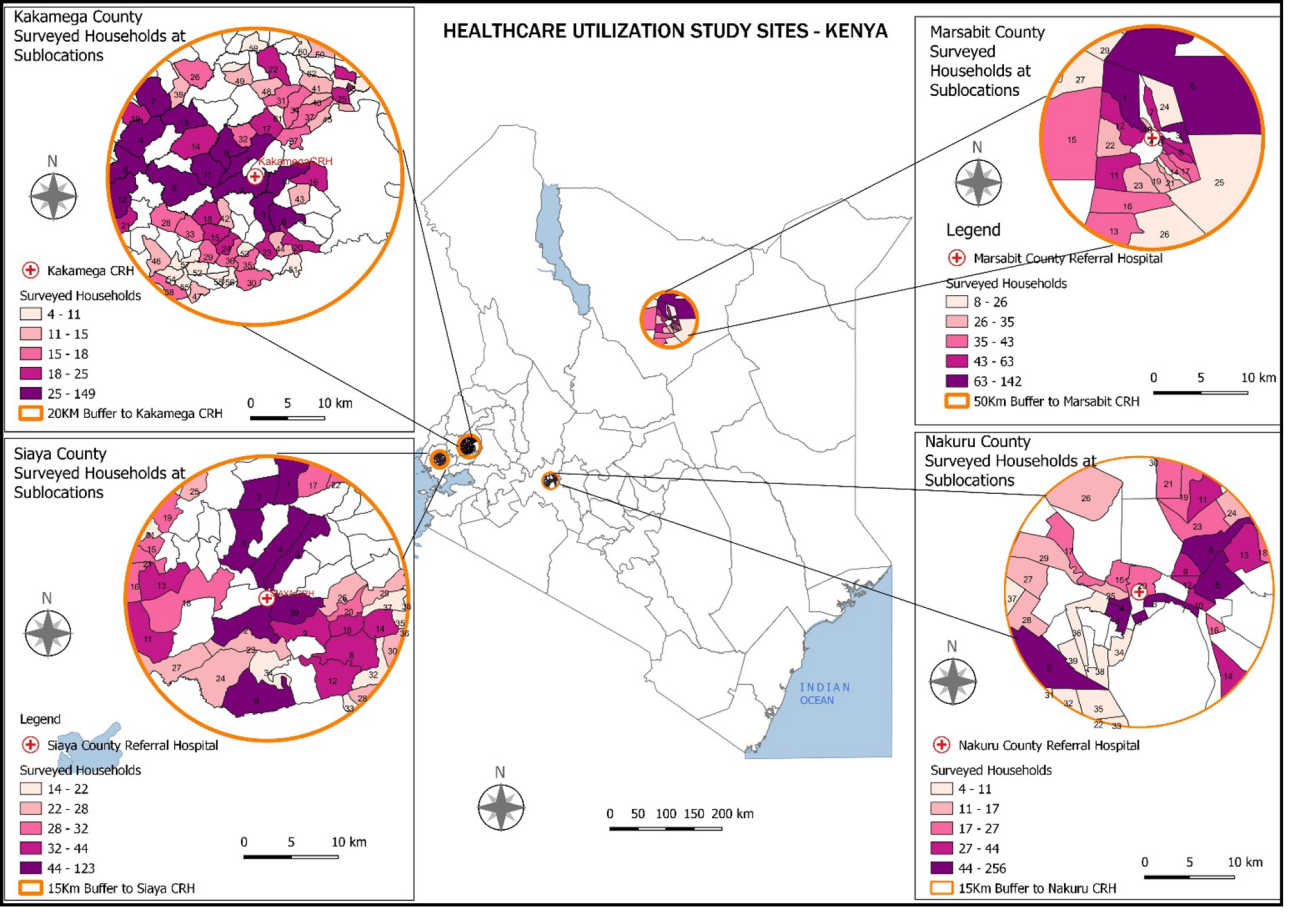
Map of Kenya showing the study locations and sample sublocations

Four diverse counties representing urban, peri-urban, rural mixed farmers, and rural pastoralist communities in Kenya (Nakuru, Kakamega, Siaya, and Marsabit respectively) were purposively selected to participate in this survey (Fig. 1). These four counties are among the eight in Kenya where sentinel surveillance sites for severe acute respiratory illness (SARI) are located [14, 15]. In 2009, the estimated population of Nakuru County was 1,603,325 of which 54% lived in urban settings [16]. Nakuru county referral hospital (NCRH) is the main referral public hospital for urban and peri-urban residents of Nakuru County. Marsabit County is a semi-arid area in the northern part of Kenya, which is dominated by pastoralists who keep large herds of cattle and camels. As of 2009, the population of Marsabit county was 291,166 of which 78% lived in rural settings [16]. Marsabit CRH (MCRH), a public hospital, is the main referral hospital in the county.

Kakamega CRH (KCRH), a public hospital, is the main referral hospital for the urban and peri-urban residents of Kakamega County in western Kenya. As of 2009, the population of Kakamega County was 1,660,651 of which 56% lived rurally [16]. The population of Siaya County, also located in western Kenya was 842,304 in 2009 of which 89% lived in rural settings [16]. Siaya CRH (SCRH) is a public hospital and serves as the main referral hospital for the county.

### Study design and sampling methods

We conducted a cross-sectional survey to determine healthcare utilization patterns and frequency of ARI and severe pneumonia using a two-stage cluster sampling procedure – first sublocation then household.

#### Catchment areas and household selection for the survey

We determined that at least 80% of the SARI patients who sought healthcare at each surveillance hospital resided in 58 sub-locations in Nakuru, 37 in Marsabit, 121 in Kakamega and 61 in Siaya (Supplementary File) [17]. Using a list of all these sub-locations, we randomly sampled (irrespective of size) a subset of sub-locations from each area for inclusion in the survey: 39/58 in Nakuru, 29/37 in Marsabit, 63/121 in Kakamega and 39/61 in Siaya. Once selected, the number of households in each of these sub-locations were allocated proportionate to the population size (PPS) based on the 2009 census.

Using the Geographic Information System (GIS) software, ArcGIS® software by Esri, we generated random spatial coordinates for the number of households that were required from each of the selected sub-locations. The survey teams located the coordinates using handheld GPS units, and the nearest household was selected for inclusion in the survey. Only households with a child aged < 5 years were included in the survey. In cases where there were no apparent households within a radius of 200 m, or there was no child aged < 5 years, the study team moved on to the next set of coordinates.

Because of the nomadic communities living in Marsabit county, a combination of random geographical coordinates (in areas where there were residential houses that could be visualized on Satellite images) and systematic sampling procedures (in rural areas with make-shift nomadic dwellings) were used to identify households to participate in the survey. For the nomadic settlements, we first generated a list of these communities and their number of households. Settlements that participated in the survey were then randomly selected. In each of the selected settlements, a sampling interval was determined by dividing its size by the number of targeted households (Supplementary File).

### Sample sizes

The sample size was powered to estimate the proportion of household respondents who had been hospitalized for an episode of pneumonia in the last year. We assumed (a) that 2.1% of the respondents would report an episode of pneumonia in the last 12 months; (b) an average of 4 persons per household [18]; (c) an estimated rate of hospitalization for pneumonia of 16%, as determined in the 2005 survey [3]; and (d) a precision of 10%. This led to a sample size of 620 households to yield at least 52 persons with self-reported pneumonia. Further assuming a design effect of two, and allowing for a nonresponse rate of 15%, the effective sample size was determined to be 1,450 households per county.

### Survey definitions

We defined a case of ARI as those who reported two or more of the following in the last 14 days preceding the survey: cough (new or worsening of chronic cough), difficulty breathing, rapid breathing, runny nose, sore throat, but were not hospitalized or recommended for hospitalization. Severe pneumonia was defined as a participant reporting an episode of respiratory illness in the previous 12 months with (i) cough and difficulty breathing for more than two days, or (ii) a physician-diagnosis of pneumonia [3, 4, 19] and (a) hospitalization or a recommendation for hospitalization by a healthcare worker, or (b) in the case of children aged < 5 years at least one danger sign, or for those > = 5 years, a “limitation” in the ability to perform routine activities. A danger sign for children < 5 years was defined as one of inability to breastfeed or drink, persistent vomiting, convulsions or seizures, loss of consciousness [3, 4]. For persons aged ≥ 5 years, a “limitation” in the ability to perform routine activities was assessed using a set of 5 questions (Supplementary File). Briefly, these questions were related to the performance of routine activities such as playing, walking, eating, self-grooming, lifting objects and were scored on a 3-point scale (“not limited”, “limited a little” or “limited a lot”). Those who reported that they were “limited a lot” in a at least two of the five questions were considered as severely ill and thus counted as cases of severe pneumonia.

Outpatient health facilities were defined as all sources of healthcare that did not admit patients for overnight stay and included both public and private ambulatory clinics. Inpatient providers included both public and private hospitals. We defined a household as a persons living together with a common cooking area [18].

### Data collection and management

Using a structured survey instrument electronically loaded on a netbook (Siaya and Kakamega) or on a tablet (Nakuru and Marsabit), trained interviewers collected household and individual data and details of episodes of respiratory disease. An adult proxy was interviewed for children < 18 years, and household members who were not present at the time of interview, including residents who died within the last 12 months. For each household member reporting an episode of ARI or pneumonia, respondents were asked detailed questions about symptoms and healthcare sought, including sources of care and whether the household member was hospitalized, or a healthcare worker had recommended hospitalization. All data were stored in a password-protected SQL database at the KEMRI offices in Kisumu and Nairobi.

### Statistical analyses

For this analysis, only data from the last episode of disease syndrome was included if more than one episode had been reported. Using principal component analysis, specifically using the factor effects derived from the first component of household goods, house construction material, source of water supply, source of cooking fuel and sanitation facility [20, 21], we generated the household wealth index as proxy for socioeconomic status (SES) stratified by county. The wealth index was categorized into quintiles; wealthy households in this study were defined as those whose wealth index was in the fourth or fifth quintile.

All analyses were conducted using Stata 15.1 software (StataCorp. 2017. Stata Statistical Software: Release 15. College Station, TX: StataCorp LLC). Multivariable survey logistic regression methods were used in the analyses to identify factors that were independently associated with healthcare seeking behaviors for respiratory illness while accounting for the survey design. Variables that were included in each of the multivariable models assessed were site (county where data were collected), sex, age, SES, and level of education of the head of the household. Other variables that were assessed included religion, household size, childbirth order, and family member status (i.e., part of the nuclear family or other relative).

## Ethical considerations

The protocol for this survey was reviewed and approved by the institutional review boards of the U.S. Centers for Disease Control and Prevention (CDC-7130), the ethical review committee of KEMRI (SSC-3667), and the institutional review board of Washington State University (IRB00000449). Written informed consent was obtained from the head of the household/main respondent prior to participation in the survey, as well as for children aged less than 13 years and household members who were not present. In addition, informed consent was obtained from adult household members who were present at the time of the survey prior to participation in the interviews. Assent was obtained from minors aged ≥ 13 years.

## Results

Between August and September 2018, we interviewed residents of 5,407 households in the four counties (Nakuru = 1,298, Marsabit = 1,353, Kakamega = 1,392 and Siaya = 1,364). Overall, there were 28,072 individuals who lived in these households and were included in the survey. Of these, 52.9% were female and 25.9% were children aged < 5 years old (Table 1). The median age of participants was 13.7 years (IQR 4.7–29.3) with only 1.6% aged 65 years. Of the participating households, 35.1% were headed by a female, 83.5% were Christian, while 14.4% were Muslim. Overall, 37.2% of household heads had attained secondary education or higher: 63.2% in Nakuru, 24.1% in Marsabit, 36.1% in Kakamega and 26.0% in Siaya County (Supplementary Table 1).

**Table 1:** Characteristics of the individuals who participated in the survey by county, Kenya, 2018

Characteristic	Nakuru (N = 6,127)	Marsabit (N = 6,999)	Kakamega (N = 7,468)	Siaya (N = 7,478)	Combined (N = 28,072)
	n (%)	n (%)	n (%)	n (%)	n (%)
Age (years)					
<2	742 (12.1)	684 (9.8)	750 (10.0)	693 (9.3)	2,869 (10.2)
2–4	906 (14.8)	1,226 (17.5)	1,119 (15.0)	1,158 (15.5)	4,409 (15.7)
5–17	1,599 (26.1)	2,207 (31.5)	2,317 (31.0)	2,580 (34.5)	8,703 (31.0)
18–49	2,545 (41.5)	2,532 (36.2)	2,777 (37.2)	2,606 (34.9)	10,460 (37.3)
50–64	260 (4.2)	238 (3.4)	360 (4.8)	314 (4.2)	1,172 (4.2)
≥65	75 (1.2)	112 (1.6)	145 (1.9)	127 (1.7)	459 (1.6)
Sex (female)	3,310 (54.0)	3,577 (51.1)	3,992 (53.5)	3,959 (52.9)	14,838 (52.9)
Education					
None	1,352 (22.1)	3,365 (48.1)	1,613 (21.6)	1,585 (21.2)	7,915 (28.2)
Pre-primary	594 (9.7)	685 (9.8)	803 (10.8)	1,028 (13.8)	3,110 (11.1)
Primary	2,037 (33.3)	2,170 (31.0)	3,488 (46.7)	3,653 (48.9)	11,348 (40.4)
Secondary	1,493 (24.4)	526 (7.5)	1,171 (15.7)	940 (12.6)	4,130 (14.7)
College/University	601 (9.8)	243 (3.5)	378 (5.1)	236 (3.2)	1,458 (5.2)
Unknown	50 (0.8)	10 (0.1)	15 (0.2)	36 (0.5)	111 (0.4)
Relationship with household head					
Head	1,297 (21.2)	1,353 (19.3)	1,392 (18.6)	1,364 (18.2)	5,406 (19.3)
Spouse	1,004 (16.4)	1,057 (15.1)	1,069 (14.3)	1,047 (14.0)	4,177 (14.9)
Son/daughter	3,077 (50.2)	4,134 (59.1)	3,843 (51.5)	4,129 (55.2)	15,183 (54.1)
Grandchild	432 (7.1)	157 (2.2)	836 (11.2)	622 (8.3)	2,047 (7.3)
Other	317 (5.2)	298 (4.3)	328 (4.4)	316 (4.2)	1,259 (4.5)
Chronic medical condition					
Any condition^a^	558 (9.1)	771 (11.0)	811 (10.9)	718 (9.6)	2,858 (10.2)
Hypertension/heart disease	156 (2.6)	138 (2.0)	221 (3.0)	156 (2.1)	671 (2.4)
Diabetes	44 (0.7)	44 (0.6)	62 (0.8)	21 (0.3)	171 (0.6)
Cancer	11 (0.2)	7 (0.1)	4 (0.1)	10 (0.1)	32 (0.1)
Arthritis	49 (0.8)	40 (0.6)	115 (1.5)	84 (1.1)	288 (1.0)
Ulcers	309 (5.0)	521 (7.4)	377 (5.1)	338 (4.5)	1,545 (5.5)
Asthma	68 (1.1)	80 (1.1)	202 (2.7)	224 (3.0)	574 (2.0)

^a^Any of the listed chronic medical conditions

### Frequency of reported episodes of respiratory illness

**Fig. 2:**
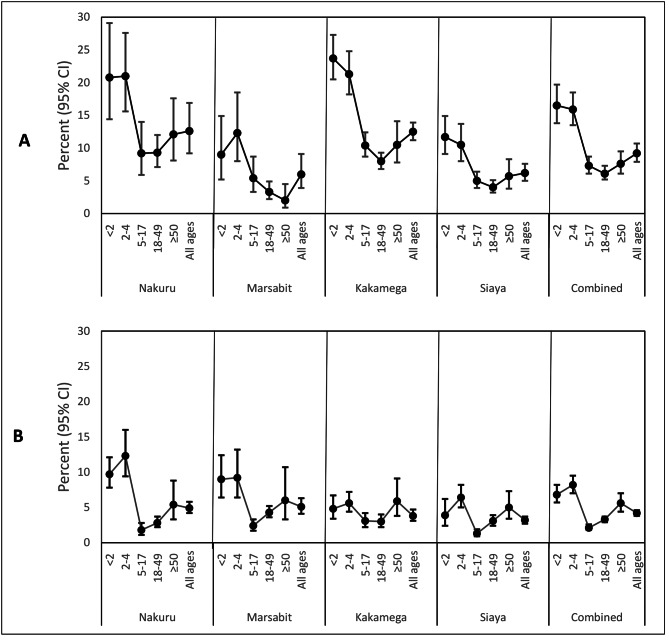
Episodes of respiratory illness. **(A)** Percent of respondents who reported an episode of acute respiratory illness in the last 14 days by county and age-category, 2018. **(B)** Percent of respondents who reported at least one episode of severe pneumonia in the last 12 months, by county and age-category, 2018

Of the survey participants, 2,614 (9.2%, 95% confidence interval [CI] 7.9–10.7%) reported at least one episode of ARI (Fig. 2 and Supplementary Tables 2 and 3). We also identified 1,159 persons of all ages (4.2%, 95% CI 3.8–4.6) who reported at least one episode of severe pneumonia in the last 12 months (Fig. 2 and Supplementary Table 2). Of all the severe pneumonia cases, 28.0% were either hospitalized or had been recommended for hospitalization by a healthcare worker when they had pneumonia. The rest (72.0%) were classified as severe cases using the study criteria defined above (31.0% children aged < 5 years who had a danger sign reported, and 41.0% persons aged ≥ 5 years who reported that they were “limited a lot” to perform routine chores).

Overall, children aged < 5 years were most likely to report at least one episode of severe pneumonia (6.8% for those aged < 2 years, and 8.2% for those aged 2–4 years) followed by persons aged ≥ 50 years (5.6%). The percentage of persons reporting severe pneumonia was similar across the four counties, except in young children < 5 years where Nakuru had significantly higher percentages compared to Kakamega (11.2% vs. 5.2%, OR = 0.43, 95% CI 0.31–0.60; p < 0.001) and Siaya (11.2 vs. 5.6%, OR = 0.47, 95% CI 0.33–0.66, p < 0.001) but not significantly higher than Marsabit (11.2% vs. 9.1%; OR = 0.80, 95% CI 0.55–1.16; p = 0.442) (Supplementary Table 3). The frequencies of episodes of severe pneumonia were comparable across the four counties for older persons (Fig. 2 and Supplementary Table 2).

### Healthcare-seeking behavior for acute respiratory illness

**Fig. 3:**
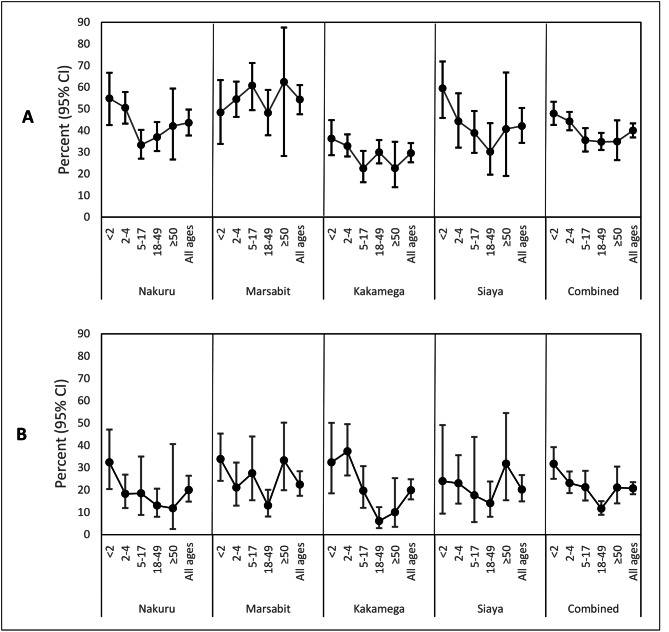
Healthcare seeking for respiratory illness. **(A)** Percent of respondents with reported cases of acute respiratory illness in the last 14 days who went to a medical health facility, by county and age-category, 2018. **(B)** Percent of respondents with severe pneumonia in the last 12 months who were hospitalized, by county and age-category, 2018

Among respondents who reported ARI within the two weeks prior to the survey, 40.0% (95% CI 36.8–43.3) went to a clinic or were seen as outpatients in a hospital, and 21.5% (95% CI 17.7–25.9) did not go to a facility but reported purchasing drugs from a pharmacy or shop. Overall, the percentage of reported ARI cases that sought care at a health facility was highest among children aged < 5 years: 47.9% among those aged < 2 years and 44.3% among children 2–4 years. The percentage of those with ARI who were seen as outpatients at a clinic or hospital was lowest in Kakamega (29.6%) and highest in Marsabit (54.3%), see Table 2; Fig. 3, and Supplementary Table 4.

**Table 2:** Healthcare seeking behavior for acute respiratory illness in the last 14 days, by county and age-category, Kenya, 2018

County	Age (years)	Number^a^	% who sought healthcare outside home*	% who sought healthcare at any health facility	% who purchased drugs at a pharmacy or shop
			% (95% CI)	% (95% CI)	% (95% CI)
Combined	<2	481	67.6 (62.9–71.9)	47.9 (42.6–53.3)	17.4 (13.2–22.5)
	2–4	714	66.9 (62.2–71.3)	44.3 (40.1–48.6)	20.7 (16.3–26.0)
	5–17	640	59.1 (54.2–63.8)	35.5 (30.3–41.1)	21.9 (17.0–27.6)
	18–49	652	59.3 (54.1–64.3)	34.8 (31.0–38.9)	24.0 (19.2–29.6)
	≥50	129	63.5 (53.8–72.2)	34.9 (26.3–44.7)	27.8 (19.9–37.4)
	**All ages**	**2,616**	**63.1 (59.3–66.6)**	**40.0 (36.8–43.3)**	**21.5 (17.7–25.9)**
Nakuru	<2	156	69.9 (64.1–75.2)	54.9 (42.5–66.7)	12.4 (4.9–28.0)
	2–4	198	67.0 (56.1–76.4)	50.5 (43.2–57.8)	14.9 (5.6–34.2)
	5–17	145	55.3 (46.2–64.1)	33.3 (27.0–40.3)	17.7 (9.7–30.2)
	18–49	236	56.5 (44.6–67.8)	37.0 (30.5–43.9)	18.7 (9.2–34.2)
	≥50	41	57.9 (46.0–68.9)	42.1 (26.6–59.4)	15.8 (6.0–35.6)
	**All ages**	**776**	**61.7 (54.3–68.6)**	**43.6 (37.7–49.7)**	**16.1 (7.7–30.9)**
Marsabit	<2	62	67.7 (54.0–79.0)	48.4 (33.8–63.3)	16.1 (5.7–38.1)
	2–4	154	72.1 (61.9–80.4)	54.5 (46.3–62.6)	14.9 (8.7–24.5)
	5–17	120	65.8 (55.7–74.7)	60.8 (49.4–71.2)	5.8 (2.7–12.3)
	18–49	83	60.2 (47.1–72.0)	48.2 (37.8–58.8)	12.0 (6.0–22.7)
	≥50	8	100.0 (- - -)	62.5 (28.2–87.6)	37.5 (12.4–71.8)
	**All ages**	**427**	**67.9 (59.5–75.3)**	**54.3 (47.5–61.0)**	**12.4 (7.2–20.5)**
Kakamega	<2	179	63.7 (55.4–71.3)	36.3 (28.6–44.8)	25.1 (18.7–32.9)
	2–4	240	61.7 (52.6–70.0)	32.9 (28.0–38.2)	27.5 (21.3–34.7)
	5–17	244	52.5 (43.1–61.7)	22.5 (16.1–30.6)	27.9 (20.5–36.7)
	18–49	227	58.6 (51.0–65.8)	30.0 (24.8–35.6)	28.6 (21.8–36.6)
	≥50	53	56.6 (39.3–72.4)	22.6 (13.8–34.8)	32.1 (19.0–48.7)
	**All ages**	**943**	**58.6 (51.3–65.6)**	**29.6 (25.3–34.2)**	**27.7 (22.7–33.3)**
Siaya	<2	84	71.4 (55.2–83.5)	59.5 (45.8–71.9)	10.7 (4.5–23.4)
	2–4	122	70.5 (60.5–78.8)	44.3 (32.1–57.2)	23.8 (13.9–37.5)
	5–17	131	69.5 (57.6–79.2)	38.9 (29.7–49.0)	29.8 (17.8–45.4)
	18–49	106	66.0 (52.3–77.5)	30.2 (19.6–43.4)	34.9 (22.6–49.6)
	≥50	27	74.1 (54.2–87.4)	40.7 (19.0–66.8)	33.3 (16.9–55.1)
	**All ages**	**470**	**69.6 (60.5–77.3)**	**42.1 (34.3–50.4)**	**26.2 (17.1–37.9)**

^a^Number who reported an episode of acute respiratory illness in the last 14 days; *Includes those who went to a health facility, pharmacy/shop, traditional healer

### Healthcare-seeking behavior for severe pneumonia

Of the 1,159 the respondents who reported at least one episode of severe pneumonia in the last 12 months, 74.2% (95% CI 70.2–77.9) sought care at a health facility, while 20.8% (95% CI 17.5–24.6) did not go to a facility but purchased drugs from a pharmacy or shop (Table 3; Fig. 3). Of those who sought care for severe pneumonia at a medical health facility, 28.9% (95% CI 25.6–32.6) were hospitalized while 7.0% (95% CI 5.4–9.1) had been recommended for hospitalization by a healthcare worker but did not get hospitalized (Nakuru = 6.8%, Marsabit = 4.4%, Kakamega = 12.9%, Siaya = 4.9%). Overall, only 20.8% (95% CI 18.2–23.6) of those who reported an episode of severe pneumonia were hospitalized. Except for Marsabit, children aged < 5 years had the highest percentage of hospitalized severe pneumonia cases. Two in every three (67.5%) of the reported cases for severe pneumonia were hospitalized in public hospitals.

**Table 3:** Healthcare seeking behavior for severe pneumonia in the last 12 months, by county and age-category, Kenya, 2018

County	Age (years)	Number^a^	% who sought healthcare outside home*	% who sought healthcare at any health facility	% who purchased drugs at a pharmacy or shop	% who were hospitalized
			% (95% CI)	% (95% CI)	% (95% CI)	% (95% CI)
Combined	<2	190	99.5 (96.1–99.9)	88.1 (82.1–92.3)	10.4 (6.5–16.3)	31.9 (25.2–39.4)
	2–4	348	97.1 (94.5–98.5)	76.3 (70.8–81.1)	19.0 (14.3–24.9)	23.4 (18.9–28.7)
	5–17	185	97.8 (93.4–99.3)	69.4 (59.1–78.1)	26.2 (18.3–35.9)	21.3 (15.4–28.7)
	18–49	344	93.8 (91.1–95.7)	68.5 (62.7–73.9)	24.3 (19.4–29.9)	11.6 (8.9–15.0)
	≥50	92	97.8 (91.3–99.5)	68.9 (59.3–77.1)	27.1 (19.2–36.7)	21.1 (14.0–30.5)
	**All ages**	**1159**	**96.7 (95.4–97.6)**	**74.2 (70.2–77.9)**	**20.8 (17.5–24.6)**	**20.8 (18.2–23.6)**
Nakuru	<2	72	100.0 (- - -)	91.2 (79.9–96.4)	6.1 (2.0–17.1)	32.4 (20.4–47.1)
	2–4	106	99.0 (92.6–99.9)	86.5 (79.3–91.5)	7.2 (3.5–14.2)	18.3 (11.9–26.9)
	5–17	27	100.0 (- - -)	85.2 (67.7–94.1)	8.0 (2.1–25.7)	18.5 (8.8–35.0)
	18–49	73	95.7 (90.9–98.0)	75.4 (63.2–84.5)	16.1 (9.3–26.5)	13.0 (8.0–20.6)
	≥50	19	100.0 (- - -)	76.5 (48.0–92.0)	23.5 (8.0–52.0)	11.8 (2.5–40.6)
	**All ages**	**297**	**98.6 (96.9–99.4)**	**84.2 (76.4–89.8)**	**10.1 (5.7–17.2)**	**20.0 (14.8–26.4)**
Marsabit	<2	59	100.0 (- - -)	93.2 (84.3–97.2)	6.8 (2.8–15.7)	33.9 (24.1–45.3)
	2–4	109	94.5 (90.0–97.0)	76.1 (66.5–83.7)	17.8 (10.5–28.6)	21.1 (13.0–32.3)
	5–17	53	98.1 (86.5–99.8)	90.4 (80.9–95.4)	6.0 (2.1–15.9)	26.9 (15.0–43.5)
	18–49	108	92.6 (87.2–95.8)	78.7 (67.5–86.8)	12.4 (6.6–21.9)	13.0 (8.1–20.1)
	≥50	21	100.0 (- - -)	81.0 (66.0–90.3)	19.0 (9.7–34.0)	33.3 (19.9–50.2)
	**All ages**	**350**	**95.7 (93.5–97.2)**	**82.1 (76.7–86.7)**	**12.5 (8.9–17.3)**	**22.3 (17.4–28.3)**
Kakamega	<2	34	97.1 (80.6–99.6)	82.4 (67.5–91.3)	15.2 (7.0–29.7)	32.4 (18.5–50.1)
	2–4	59	98.3 (87.9–99.8)	76.3 (64.5–85.0)	22.4 (13.9–34.1)	37.3 (26.5–49.5)
	5–17	71	97.2 (84.4–99.5)	53.5 (36.5–69.7)	43.3 (29.0–58.8)	19.7 (12.0–30.7)
	18–49	83	91.5 (85.2–95.2)	52.4 (40.9–63.7)	41.1 (29.7–53.5)	6.1 (2.9–12.3)
	≥50	30	93.3 (75.1–98.5)	66.7 (47.4–81.6)	23.1 (10.3–43.8)	10.0 (3.5–25.3)
	**All ages**	**277**	**95.3 (92.1–97.2)**	**63.0 (53.6–71.6)**	**32.3 (24.7–40.9)**	**19.9 (15.8–24.8)**
Siaya	<2	25	100.0 (- - -)	80.0 (60.5–91.3)	20.0 (8.7–39.5)	24.0 (9.4–49.1)
	2–4	74	95.9 (84.3–99.0)	63.5 (49.4–75.6)	32.9 (20.1–48.7)	23.0 (13.9–35.6)
	5–17	34	94.1 (81.0–98.4)	58.8 (42.4–73.5)	35.5 (19.0–56.3)	17.6 (5.6–43.8)
	18–49	80	96.2 (85.1–99.1)	65.4 (53.0–76.0)	30.1 (20.1–42.5)	14.1 (8.0–23.8)
	≥50	22	100.0 (- - -)	54.5 (37.5–70.6)	42.9 (26.1–61.4)	31.8 (15.4–54.5)
	**All ages**	**235**	**96.6 (91.6–98.6)**	**64.4 (58.1–70.2)**	**31.8 (25.2–39.2)**	**20.2 (14.9–26.7)**

^a^Number who reported an episode of severe pneumonia in the last 12 months; *Includes those who went to a health facility, pharmacy/shop, traditional healer

### Factors associated with healthcare seeking behavior for acute respiratory illness

In the multivariable model that included sex, age, level of education of the head of the household, and the households’ socio-economic status, being a young child aged < 5 years was significantly associated with seeking healthcare for ARI in a hospital or clinic compared to persons aged 5–49 years (46.3% vs. 35.7%; adjusted odds ratio [aOR] = 1.55, 95% CI 1.31–1.85; p < 0.001) but similar between those aged 5–49 years and those aged ≥ 50 years (aOR = 1.05, 95% CI 0.71–1.55; p = 0.795) (Table 4). Children aged < 5 years were also less likely to use medications purchased over the counter from a pharmacy or shop (self-medication) for an episode of ARI compared to those aged 5–49 years (19.3% vs. 22.8%; aOR = 0.78, 95% CI 0.64–0.96; p = 0.018). Respondents with ARI who were from wealthier households (SES in 4th or 5th quintile) were more likely to seek medical care at a private health facility compared to their poorer counterparts (10.9% vs. 5.5%; aOR = 2.06, 95% CI 1.33–3.19; p = 0.001). Similarly, residents of wealthier households were more likely to report self-medication for ARI compared to their counterparts in less wealthy households (27.9% vs. 17.6%; aOR = 1.82, 95% CI 1.34–2.46; p < 0.001). Whereas females in Marsabit had a significantly higher likelihood of seeking healthcare in a hospital or clinic for an episode of ARI compared to their male counterparts (61.4% vs. 51.7%; aOR = 1.50, 95% CI 1.02–2.22; p = 0.041), there was no such gender difference in the other counties (Supplementary Table 5).

**Table 4:** Factors associated with healthcare-seeking behavior for acute respiratory illness (all counties), Kenya, 2018

Characteristic		Number^a^	Went to any health facility (public or private)			Went to a private health facility			Purchased drugs from a pharmacy/shop		
			%	Adjusted Odds Ratio* (95% CI)	p-value	%	Adjusted Odds Ratio* (95% CI)	p-value	%	Adjusted Odds Ratio* (95% CI)	p-value
Sex	Male	1,230	40.0	Ref		8.1	Ref		22.5	Ref	
	Female	1,384	41.0	1.11 (0.93–1.32)	0.253	7.0	0.86 (0.64–1.18)	0.352	20.5	0.86 (0.71–1.03)	0.107
Age (years)	< 5 years	1,194	46.3	1.55 (1.31–1.85)	< 0.001	8.0	1.02 (0.73–1.42)	0.915	19.3	0.78 (0.64–0.96)	0.018
	5–49 years	1,291	35.7	Ref		7.1	Ref		22.8	Ref	
	≥ 50 years	129	34.9	1.05 (0.71–1.55)	0.795	7.1	1.15 (0.58–2.28)	0.684	27.8	1.12 (0.72–1.74)	0.608
Education level of head of household^b^	Low	1,656	40.8	Ref		5.1	Ref		21.4	Ref	
	High	958	40.1	0.99 (0.80–1.21)	0.892	11.8	1.69 (1.20–2.39)	0.003	21.6	0.92 (0.73–1.16)	0.488
Household socioeconomic status^c^	Low	1,645	40.7	Ref		5.5	Ref		17.6	Ref	
	High	969	40.1	0.98 (0.80–1.19)	0.820	10.9	2.06 (1.33–3.19)	0.001	27.9	1.82 (1.34–2.46)	< 0.001

^a^Number who reported an episode of acute respiratory illness in the 14 days preceding the survey; ^b^Level of education for the head of the household (Low = Primary level or none, High = Secondary level or higher); ^c^Household socio-economic status (Low = 1–3 quintile, High = 4–5 quintile). *Adjusted for site (county) and all variables listed

### Factors associated with healthcare seeking behavior for severe pneumonia

Children aged < 5 years were significantly more likely to seek healthcare in a hospital or clinic for an episode of severe pneumonia compared to respondents aged 5–49 years (80.1% vs. 66.7%; aOR = 1.74, 95% CI 1.27–2.38; p = 0.001) (Table 5). Similarly, children aged < 5 years were more likely to be hospitalized for severe pneumonia compared to persons aged 5–49 years (25.6% vs. 16.6%; aOR = 1.64, 1.15–2.34; p = 0.006). The odds of hospitalization were similar between persons aged 5–49 years and those aged ≥ 50 years (16.6% vs. 24.7%; aOR = 1.71, 95% CI 0.98–2.99; p = 0.060). Overall, females were less likely than males to report hospitalization for severe pneumonia (18.5% vs. 25.1; aOR = 0.70, 95% CI 0.52–0.94; p = 0.019), an association that was statistically significant in Marsabit county where 29.8% of the males were hospitalized with severe pneumonia compared to 18.3% females (aOR = 0.49, 95% CI 0.28–0.84; p = 0.013) (Supplementary Table 6). In Marsabit, respondents from households with a higher SES (4th or 5th quintile) were more likely to report hospitalization for an episode of severe pneumonia (28.7% vs. 19.8%; aOR = 1.66, 95% CI 1.06–2.58; p = 0.027) compared to households with lower SES.

**Table 5:** Factors associated with healthcare-seeking behavior for severe pneumonia in the last 12 months (all counties), Kenya, 2018

Characteristic		Number^a^	Went to any health facility (public of private)			Went to a private health facility			Hospitalized		
			%	Adjusted Odds Ratio* (95% CI)	p-value	%	Adjusted Odds Ratio* (95% CI)	p-value	%	Adjusted Odds Ratio* (95% CI)	p-value
Sex	Male	561	74.7	Ref		22.9	Ref		25.1	Ref	
	Female	598	72.5	1.01 (0.77–1.31)	0.957	24.1	1.16 (0.83–1.62)	0.381	18.5	0.70 (0.52–0.94)	0.019
Age (years)	< 5 years	538	80.1	1.74 (1.27–2.38)	0.001	28.4	1.67 (1.16–2.39)	0.006	25.7	1.64 (1.15–2.34)	0.006
	5–49 years	529	66.7	Ref		17.6	Ref		16.6	Ref	
	≥ 50 years	92	69.1	1.23 (0.78–1.94)	0.377	24.7	1.59 (0.88–2.88)	0.126	24.7	1.71 (0.98–2.99)	0.060
Education level of head of household^b^	Low	738	71.9	Ref		18.0	Ref		20.5	Ref	
	High	421	76.6	1.18 (0.84–1.67)	0.335	33.2	1.48 (1.02–2.16)	0.038	23.9	1.24 (0.89–1.73)	0.200
Household socioeconomic status^c^	Low	692	73.5	Ref		20.1	Ref		19.7	Ref	
	High	467	73.7	0.92 (0.67–1.27)	0.618	27.1	1.36 (0.88–2.11)	0.160	23.9	1.16 (0.83–1.61)	0.376

^a^Number who reported an episode of severe pneumonia in the last 12 months; ^b^Level of education for the head of the household (Low = Primary level or none, High = Secondary level or higher); ^c^Household socio-economic status (Low = 1–3 quintile, High = 4–5 quintile). *Adjusted for site (county) and all variables listed

We explored other variables - religion, household size, childbirth order, family member status (whether one was a nuclear family member or not), and distance of the household to the nearest road - to assess if there was an association with healthcare seeking behavior for respiratory illness but found no significant associations, see Supplementary Tables 7 and 8.

## Discussion

We conducted a HUS in four counties of Kenya that represented the diversity of the Kenyan population, to determine healthcare seeking behavior for respiratory illness. The trends of healthcare seeking were similar across different age groups, gender, and socio-economic status among the urban, peri-urban, and rural populations of the country including the nomadic communities inhabiting the remote arid regions. Our study found that three in every four persons with severe pneumonia sought healthcare at a health facility, either as inpatients or outpatients, which suggests that facility-based surveillance for severe respiratory illness may be reliable to capture disease burden, which varied by age, in the populations. However, we found that only 18–24% of respondents of all ages with severe pneumonia were hospitalized. Healthcare seeking for mild respiratory illness as an outpatient was generally low (37–43%), with substantial levels (18–26%) of reported cases of self-medication (i.e., purchased drugs from a pharmacy or drug shop).

For both ARI and severe pneumonia cases, our study found a significantly higher percentage of seeking care in the hospital or clinic among young children aged < 5 years compared to persons who were older. Similar findings have been reported in other studies [3, 4, 8]. One reason for higher healthcare utilization in children is that the medical costs in public hospitals in Kenya are waived for children < 5 years [22]. Similar to other studies in Kenya [3, 23], we found that male respondents in all four counties were more likely to be hospitalized, with statistically significant differences compared to females observed in Marsabit. The high percentage of hospitalization for respiratory illness among male respondents in Marsabit may be attributed to delayed healthcare seeking and therefore the development of more severe illness thus requiring hospitalization. Indeed, our findings also showed that a significantly higher percentage of female respondents in Marsabit had sought healthcare for respiratory illness as outpatients compared to male respondents.

We found that despite 70–78% of persons with severe pneumonia seeking care at a medical health facility, only 26–33% were hospitalized and that an additional 5–9% who were recommended for hospitalization did not go. Overall, when we included all cases in the community, our study found that only 18–24% were hospitalized when they had an episode of severe pneumonia. It is possible that healthcare workers, perhaps because of limited hospital bed capacities, have high thresholds for recommending hospitalization and prefer to treat as outpatients. Other factors such as inability to pay for the hospital fees, particularly among older patients who receive no waivers from government [22], may be the reasons why some patients who are recommended for hospitalization opt out.

Most (70–78%) of those who reported severe pneumonia had sought healthcare at a medical health facility. On the contrary, only 37–43% of those with ARI had sought healthcare at a medical health facility. Healthcare seeking for severe pneumonia and ARI was comparable to findings reported in a similar study conducted in Siaya in 2005 [3]. Overall, these findings suggest that outpatient surveillance for respiratory illness in our study populations would capture less than half of the disease burden. To determine the true burden of severe respiratory disease in the community, our study incorporated additional questions for indicators of health for all respondents who reported pneumonia in the last year as a criterion to determine those who had severe illness. Further studies are warranted to validate the performance of this criteria in discriminating severity of illness.

Kenya has a mixed health financing system through revenues collected by the government (national and county – 37%), donor funding (23%), out-of-pocket payments by patients (26%) and other private sources [24]. In 2016, the overall public expenditure on health as a percentage of GDP was estimated at 2.2%. Less than 20% of the population in Kenya have a health insurance [24]. Therefore, most of the population must pay for the healthcare out-of-pocket. A recent costing study found that families spent US$ 20 (US$ 21 for children < 5 years and US$ 17 for persons aged ≥ 5 years) for outpatient visits, and US$ 118 (US$ 114 for children < 5 years and US$ 137 for persons aged ≥ 5 years) for hospitalizations associated with a single episode of influenza infection. The cost of hospitalization for an episode of respiratory illness represented 40–60% of the household monthly income [25]. It is therefore conceivable that families would opt not to be hospitalized, even if a clinician determines otherwise, while others seek to use alternative treatment options altogether, such self-medication by purchasing drugs over the counter, especially if they considered their illness not to be serious enough to warrant medical care at a medical facility [10]. Kenya recently conducted a pilot of universal healthcare coverage (UHC) in four counties (Kisumu, Isiolo, Machakos and Nyeri) which is aimed at cushioning citizens against the impoverishing effects of out-of-pocket healthcare payments [26]. By removing user fees, the UHC is expected to increase access, and address inequity in access, to healthcare services.

Our study had several limitations. First, self-reporting of ARI and pneumonia was not verified by medical records and may result in recall bias especially as we asked for episodes of severe pneumonia during the last 12 months. Second, severe pneumonia classification based on limitation of activities may have misclassified non-lower respiratory illness into the severe pneumonia category. Third, older populations, particularly those aged ≥ 50 years, were underrepresented in our sampled households because we targeted households that had at least one child aged < 5 years. As such findings from this survey may not necessarily be generalizable to households that did not have young children. Fourth, we didn’t assess the possible effect distance from the health facility on healthcare seeking for respiratory illness. Fifth, our sample size was not powered to the age categories that we present here and may have affected the age-specific comparisons and precision of healthcare utilization estimates. Last, we conducted our study over a two-month period, and specifically for ARI, we only assessed episodes that occurred within 14 days that preceded the survey. If the survey coincided with seasonal events such as planting or harvesting, it is possible that we underestimated healthcare seeking for ARI especially if community members would prioritize those seasonal activities. However, this may not necessarily have affected our estimates for severe respiratory illness as those were assessed over the last 12 months.

## Conclusion

Our findings suggest that hospital-based surveillance for severe respiratory illness captured less than one quarter of the disease burden in the community in Kenya. Data from community household surveys need to be considered, particularly in low- and middle-income countries, to provide useful adjustment factors to account for low healthcare seeking behaviors and utilization of hospital resources for severe respiratory illness.

## Electronic supplementary material

Below is the link to the electronic supplementary material.

Supplementary Material 1

SupplementaryMaterial 2

## Data Availability

All the data used in the preparation of this manuscript have been provided in the manuscript and supplementary files. The underlying data used to prepare the tables and figures presented in this manuscript may be requested and can be shared subject to the approval by the relevant IRB institution. Requests for additional data should be directed to Dr Gideon Emukule (Email: uyr9@cdc.gov) or Dr Godfrey Bigogo (Email: GBigogo@kemri.go.ke).
