# Supplementary material for: Healthcare-seeking behavior for respiratory illnesses in Kenya: implications for burden of disease estimation
Source: BMC Public Health. 2023 Feb 16;23:353. doi: 10.1186/s12889-023-15252-3 (PMC9936639; doi:10.1186/s12889-023-15252-3)
Supplement: Supplementary file 1 — Supplementary Material 1 [file 12889_2023_15252_MOESM1_ESM.docx]

**Supplementary Methods**

1. **Defining hospital catchment areas for the survey**

The ongoing surveillance for severe acute respiratory illness (SARI) at Kakamega, Nakuru, and Siaya county referral hospitals (CRH) provided a line list of in-patients including their place of residence by county, location, and village/estate. In Marsabit, we conducted a record review of patients who were admitted at Marsabit CRH with a respiratory illness, over a period of 2 years, to identify their areas of residence. To identify the catchment areas of these hospitals, we used data from patients who were hospitalized with SARI over the last two years. Using these data, we matched the residence data to the corresponding sub-locations as per the 2009 census data. Data from the 2009 census [[1](#_ENREF_1)], are available, with corresponding mapped shape files at the sub-location level (the second lowest administrative level in Kenya). The data elements associated with the shapefiles are population size (disaggregated by sex), population density, and number of households (see definition provided further below) as per the 2009 census. Considering that some of the sub-locations had since been renamed or sub-divided, we enlisted help of the local residents and administrative leaders to ensure that residence data were appropriately matched. In descending frequency, we identified the sub-locations where ≥80% of the SARI patients resided [[2](#_ENREF_2)], and determined a radial distance, from the hospital and covering these sub-locations, as the catchment area for the hospital. The radial distances varied across the study hospitals and were determined as 15 kilometers (km) for Nakuru, 15 km for Siaya, 20 km for Kakamega and 50 km for Marsabit. Using the list of sub-locations in the catchment areas of the respective surveillance hospitals, a random sample of sub-locations was selected for inclusion in the survey: 39/58 in Nakuru, 29/37 in Marsabit, 63/121 in Kakamega and 39/61 in Siaya.

1. **Selection of households included in the survey**

The Kenya National Bureau of Statistics (KNBS) has the shape files (at the sub-location level) that also contain the 2009 population census data. Using the Geographic Information System (GIS) software, ArcGIS, we generated random spatial coordinates corresponding to the number of households required from each sub-location included in the survey. The field teams used a GPS sensor to locate these randomly generated GPS coordinates. The households closest to the GPS coordinates were approached for enrolment in the survey. If no households were apparent, the next nearest GPS coordinate was located, and the nearest household approached for participation in the survey. An interviewer approached the adult female or male head of the household and requested consent for the household’s participation in the survey. If consent was not given, the next closest household was approached. The summary of steps followed to identify households that were included in the survey is shown in Figure 1.

**Figure 1:** Summary of the steps to be used to select households included in the survey

**Step 1:** Defined the catchment areas of the hospitals (where at least 80%of the SARI patients resided) and made a list of the catchment sub-locations for each hospital.

**Step 2:** Randomly selected the sub-locations to be included in the survey from each of the 4 study catchment areas.

**Step 3:** Determined and selected the number of households to be included from each hospital’s catchment population proportionate to the population size.

**Step 4:** Randomly generated a list of GPS coordinates, representing each household to be sampled from each selected sub-location, using the ArcGIS software.

**Step 5:** Navigate to the provided GPS coordinates and enroll the closest household if it has a child aged<5 years.

***Household selection procedures in Marsabit County***

Because of the nomadic communities living in Marsabit county, a combination of random geographical coordinates and systematic sampling procedures were used to identify households that participated in the survey. Systematic sampling procedures were used to identify households that participated in the survey in 6 sub-locations (434 households) out of the 29 sub-locations (1,353 households) that participated in the survey in Marsabit county. These sublocations included Gar-Qarsa, Quachacha, Kambinye, Kargi, Medate Kuro and Shura

*Systematic sampling approach:*

This approach was used to identify households that were not visualized on google maps. We line listed names of settlements and the corresponding number of households in each settlement. Settlements that participated in the survey were randomly selected. In each of the selected settlements, a sampling interval was calculated by dividing total size of the settlement (total number of households) by the desired target number of households to be sampled. On the day of sampling in a settlement, a research assistant would spin a bottle while standing roughly in the middle of the settlement/*manyatta* to determine the direction of movement from the central point. The first household to be sampled was picked by random selection of a number between 1 and the value of the sampling interval. This was done by random drawing of a number from a bag. Subsequent households were enrolled in the same direction using the sampling interval (For all teams, sampling interval was between 2-4). If a household was ineligible, the team would sample the house located to the left or right of the ineligible household. In case study team did not attain the target in the first round of sampling, they would decide to turn right or left at the village boundary and continue sampling until the desired target is achieved (See Figure 2 below)

**Figure 2**: Illustration systematic sampling in Marsabit

|  | Village boundary |
| --- | --- |
|  | Starting point |
|  | Direction of sampling |
|  | Household |

1. **Calculating distances to the nearest road**

To explore the potential effect of accessibility to the health facility and how that might be associated with healthcare seeking for respiratory illness, we estimated the shortest distances from the study households to the nearest road. We obtained roads data shapefile from the Kenya Roads Board. This is the official roads network database for Kenya with various roads categories. The data were collected through a Road Inventory and Conditions Survey (RICS) in 2018 and are updated regularly [[3](#_ENREF_3), [4](#_ENREF_4)]. We re-projected the household and roads data layers from WGS 84 Geographic Coordinate System in degree decimals to WGS 84 Universal Transverse Mercator (UTM) in meters, a two-point equidistant projection system suitable for small areas, using QGIS software. We used the Shortest-Line-Between-Features tool in QGIS to calculate the distance in meters in the re-projected UTM coordinate system between the households and the roads.

1. **Criteria for defining severe pneumonia**

Since many cases of severe pneumonia may occur and not seek health care, and we wanted to measure the true burden of severe pneumonia, we defined severe pneumonia for children <5 years old as those who met the pneumonia case definition (cough and difficulty breathing for more than two days, or a physician-diagnosis of pneumonia) and had any of the following signs or symptoms reported: inability to breastfeed or drink, persistent vomiting, convulsions or seizures, loss of consciousness, decreased activity (unable to play) [[5](#_ENREF_5), [6](#_ENREF_6)]. For those ≥5 years we asked five additional questions to assess severity of illness as perceived by the respondent (Table 1).

Table 1: Additional questions asked of respondents who reported an episode of pneumonia to assess those who had severe illness.

| ***Questions for children aged 5-17 years*** | |
| --- | --- |
| 1 | Was (NAME) limited in doing things that takes a lot of energy (e.g., playing soccer, running, riding a bike) due to the illness? |
| 2 | During the illness, were you/spouse LIMITED in the amount of time YOU had for your own needs because of your (NAME’s) physical health? |
| 3 | Was (NAME) limited in walking for 5 minutes (walking to the neighboring home) due to the illness? |
| 4 | Was (NAME) limited taking care of him/herself (e.g., eating, dressing, bathing, or going to the toilet) due to the illness? |
| 5 | During the illness, were you/spouse LIMITED in the amount of time YOU had for your own needs because of your (NAME’s) physical health? |
| ***Questions for persons aged ≥18 years*** | |
| 1 | Was (NAME) limited in walking for 5 minutes (walking to the neighboring home) due to the illness? |
| 2 | Was (NAME) limited taking care of him/herself (e.g., eating, dressing, bathing, or going to the toilet) due to the illness? |
| 3 | Was (NAME) limited in doing vigorous activities (e.g., running, lifting heavy objects, and digging, pushing a handcart) due to the illness? |
| 4 | Was (NAME) limited in doing moderate activities (e.g., moving a table, sweeping, or making one’s bed) due to the illness? |
| 5 | Was (NAME) limited in in lifting or carrying objects (e.g., basket of groceries/cereals, bucket of water) due to the illness |

Responses to these questions were “Limited a lot”, “Limited a little” or “Not limited”. If the respondent answered “limited a lot” in at least 3/5 questions, the person was considered to have been severely ill.

Of the respondents aged ≥5 years that the study considered as severe pneumonia cases, 66% reported limited ability in in bending, lifting, or stooping, while 45% reported limited ability in eating, dressing, bathing, or going to the toilet (Table 2). Further, among those aged 5-17 years, 75% had limited ability in activities that took a lot of energy such as playing and running. Among severe pneumonia reported cases aged ≥18 years, 55% were limited a lot in carrying out activities such as making the bed. Overall, we determined that 67% of those persons aged ≥5 years who reported hospitalization for pneumonia in the last year also reported being “limited a lot” in at least two or more of the five activities assessed.

**Table 2:** Distribution of the indicators of severity among those who reported pneumonia and were classified as severe pneumonia cases, 2018

| Characteristic | Children <5 years (N=538) |  | Persons ≥5 years (N=545) |
| --- | --- | --- | --- |
|  | n (%) |  | n (%) |
| **Had been hospitalized or recommended for hospitalization** | 142 (26) |  | 99 (18) |
| **Reported symptoms indicative of severe illness (children <5 years)** |  |  |  |
| Unable to breastfeed or drink | 364 (68) |  | NA |
| Persistent vomiting | 136 (25) |  | NA |
| Convulsions or seizures | 72 (14) |  | NA |
| Loss of consciousness | 51 (9) |  | NA |
| Decreased activity | 302 (56) |  | NA |
| **Additional questions to assess severity of illness (only persons aged ≥5 years)** |  |  |  |
| Limited a lot in doing things that takes a lot of energy (e.g., playing  soccer, running) due to the illness (5-17 years) | NA |  | 132/177 (75) |
| Limited a lot in walking for 5 minutes due to the illness (≥5 years) | NA |  | 286 (53) |
| Limited a lot in bending, lifting, or stooping due to the illness (≥5 years) | NA |  | 360 (66) |
| Limited a lot taking care of him/herself (e.g., eating, dressing, bathing,  or going to the toilet) due to the illness (≥5 years) | NA |  | 246 (45) |
| Limited a lot in doing moderate activities (e.g., sweeping or making  one’s bed) due to the illness (≥18 years) | NA |  | 203/369 (55) |
| Limited a lot in lifting or carrying objects (e.g., basket of \  groceries/cereals, bucket of water) due to the illness (≥18 years) | NA |  | 251/369 (67) |
| Limited a lot in the amount of time caretaker had for own needs because  of child’s illness (5-17 years) | NA |  | 93/177 (52) |

Using the questions shown in Table 1 above to assess severity of illness among respondents aged ≥5 years who reported pneumonia,

**Table 3:** List of sublocations and number of households that participated in the survey in Nakuru County

| Sublocation | | Total households | Sampled Households |  | Sublocation | | Total households | Sampled Households |
| --- | --- | --- | --- | --- | --- | --- | --- | --- |
| 1 | Kaptembwo | 23200 | 256 |  | 23 | Thayu | 1352 | 17 |
| 2 | Njoro | 10149 | 118 |  | 24 | Wendo | 1395 | 16 |
| 3 | Langalanga | 9674 | 93 |  | 25 | Viwandani | 1297 | 15 |
| 4 | Githima | 5182 | 64 |  | 26 | Menengai R | 1324 | 15 |
| 5 | Murunyu | 5068 | 61 |  | 27 | Ngata | 964 | 14 |
| 6 | Baharini | 4829 | 56 |  | 28 | Ogilgei | 879 | 14 |
| 7 | Free-Area | 5070 | 55 |  | 29 | Kamungei | 884 | 13 |
| 8 | Kabatini | 4189 | 47 |  | 30 | Ndungiri | 1022 | 13 |
| 9 | Rurii | 2738 | 40 |  | 31 | Gichobo | 661 | 10 |
| 10 | Kiratina | 4239 | 35 |  | 32 | Naishi | 801 | 8 |
| 11 | Bahati | 2663 | 33 |  | 33 | Sinendet | 589 | 8 |
| 12 | Workers | 2661 | 33 |  | 34 | Park View | 503 | 6 |
| 13 | Mugwathi | 2361 | 29 |  | 35 | Pwani | 457 | 6 |
| 14 | Kongasis | 2309 | 28 |  | 36 | Ingobor | 362 | 5 |
| 15 | London | 3315 | 27 |  | 37 | Milimani | 390 | 5 |
| 16 | Muguga | 1370 | 27 |  | 38 | Kelelwet | 342 | 4 |
| 17 | Olive | 1671 | 23 |  | 39 | Subuku | 468 | 4 |
| 18 | Dundori | 1908 | 23 |  |  |  |  |  |
| 19 | Mutukanio | 1799 | 20 |  |  |  |  |  |
| 20 | Milimani B | 1640 | 19 |  |  |  |  |  |
| 21 | Wanyororo | 1523 | 19 |  |  |  |  |  |
| 22 | Lare | 1567 | 19 |  |  |  |  |  |

**Table 4:** List of sublocations and number of households that participated in the survey in Kakamega County

| **Sublocation** | | **Total households** | **Sampled Households** |  | **Sublocation** | | **Total households** | **Sampled Households** |  | **Sublocation** | | **Total households** | **Sampled Households** | |
| --- | --- | --- | --- | --- | --- | --- | --- | --- | --- | --- | --- | --- | --- | --- |
| 1 | Sichilayi | 10,475 | 149 |  | 23 | Museno | 1,365 | 20 |  | 45 | Buyangu | 987 | 15 |  |
| 2 | Shirere | 7,738 | 111 |  | 24 | Malinya | 1,278 | 19 |  | 46 | Mwikalikha | 1,095 | 15 |  |
| 3 | Shidodo | 2,338 | 40 |  | 25 | Kakunga | 1,333 | 19 |  | 47 | Kaluni | 961 | 14 |  |
| 4 | Isongo | 2,674 | 39 |  | 26 | Nambacha | 1,253 | 18 |  | 48 | Lunyinya | 998 | 14 |  |
| 5 | Shibuli | 2,417 | 35 |  | 27 | Lubao | 1,227 | 18 |  | 49 | Lukume | 995 | 14 |  |
| 6 | Shingodo | 2,532 | 35 |  | 28 | Mutaho | 1,304 | 18 |  | 50 | Shivikhwa | 907 | 13 |  |
| 7 | Lusumu | 2,428 | 35 |  | 29 | Lunerere | 1,311 | 18 |  | 51 | Shiveye | 750 | 11 |  |
| 8 | Makunga | 2,250 | 32 |  | 30 | Ivonda | 1,320 | 18 |  | 52 | Shabwali | 752 | 11 |  |
| 9 | Murumba | 2,104 | 31 |  | 31 | Shamoni | 1,236 | 18 |  | 53 | Lirhembe | 687 | 10 |  |
| 10 | Shiswa | 2,144 | 30 |  | 32 | Shirakalu | 1,173 | 17 |  | 54 | Emuruba | 684 | 10 |  |
| 11 | Shiyunzu | 1,919 | 28 |  | 33 | Shikulu | 1,195 | 17 |  | 55 | Munjiti | 706 | 10 |  |
| 12 | Shitari | 1,879 | 27 |  | 34 | Chevoso | 1,118 | 17 |  | 56 | Shanjetso | 605 | 9 |  |
| 13 | Shinoyi | 1,764 | 26 |  | 35 | Makhokho | 1,111 | 16 |  | 57 | Shibuname | 591 | 9 |  |
| 14 | Esumeyia | 1,818 | 25 |  | 36 | Shitoli | 1,100 | 16 |  | 58 | Shishejeri | 637 | 9 |  |
| 15 | Shivagala | 1,624 | 24 |  | 37 | Sasala | 1,079 | 16 |  | 59 | Burundu | 574 | 7 |  |
| 16 | Mukulusu | 1,753 | 24 |  | 38 | Ematsasi | 1,239 | 16 |  | 60 | Musungu | 490 | 7 |  |
| 17 | Indangalasia | 1,566 | 23 |  | 39 | Mukweso | 1,053 | 15 |  | 61 | Ifwetere | 466 | 7 |  |
| 18 | Shiseso | 1,506 | 22 |  | 40 | Shambrere | 1,008 | 15 |  | 62 | Mutingongo | 499 | 7 |  |
| 19 | Malaha | 1,538 | 22 |  | 41 | Shilongo | 1,058 | 15 |  | 63 | Fuvale | 365 | 5 |  |
| 20 | Lugose | 1,280 | 21 |  | 42 | Malimili | 1,034 | 15 |  | 64 | Shimuli | 310 | 4 |  |
| 21 | Ebushitinji | 1,435 | 21 |  | 43 | Itenyi | 1,006 | 15 |  |  |  |  |  |  |
| 22 | Shikutse | 1,366 | 20 |  | 44 | Shirulu | 1,037 | 15 |  |  |  |  |  |  |

**Table 5:** List of sublocations and number of households that participated in the survey in Siaya County

| Sublocation | | Total households | Sampled Households |  | Sublocation | | Total households | Sampled Households |
| --- | --- | --- | --- | --- | --- | --- | --- | --- |
| 1 | Ugunja | 2398 | 78 |  | 23 | Randogo | 815 | 28 |
| 2 | Nyandiwa | 2028 | 68 |  | 24 | Mur-Malanga | 815 | 28 |
| 3 | Hono | 1634 | 57 |  | 25 | Siranga | 820 | 28 |
| 4 | Komolo | 1513 | 52 |  | 26 | Umala | 811 | 27 |
| 5 | Koyeyo | 1320 | 48 |  | 27 | Bar Olengo | 682 | 24 |
| 6 | Nyajuok | 1333 | 45 |  | 28 | Kaudha East | 783 | 24 |
| 7 | Ngunya | 1294 | 45 |  | 29 | Malunga West | 671 | 23 |
| 8 | Masumbi | 1259 | 44 |  | 30 | Dienya East | 674 | 23 |
| 9 | Bar-Agulu | 1249 | 42 |  | 31 | Kalkada Uradi | 678 | 21 |
| 10 | Mur_Ngiya | 1187 | 41 |  | 32 | Dienya West | 608 | 19 |
| 11 | Kandenge | 1124 | 38 |  | 33 | Kaudha West | 563 | 18 |
| 12 | Nyangoma | 1045 | 36 |  | 34 | Bar Osimbo | 502 | 16 |
| 13 | Gangu | 940 | 33 |  | 35 | Wagai West | 465 | 16 |
| 14 | Ulamba | 973 | 33 |  | 36 | Wagai East | 510 | 15 |
| 15 | Sigoma Uranga | 994 | 32 |  | 37 | Malunga Central | 449 | 14 |
| 16 | Kaugagi Hawinga | 967 | 32 |  | 38 | Malunga East | 396 | 14 |
| 17 | Got Osimbo | 891 | 32 |  | 39 | Karapul | 3795 | 123 |
| 18 | Ojwando 'A' | 886 | 30 |  |  |  |  |  |
| 19 | Komenya Kowala | 857 | 30 |  |  |  |  |  |
| 20 | Olwa | 835 | 29 |  |  |  |  |  |
| 21 | Kaugagi Udenda | 834 | 29 |  |  |  |  |  |
| 22 | Mungao | 853 | 29 |  |  |  |  |  |

**Table 6:** List of sublocations and number of households that participated in the survey in Marsabit County

| **Sublocation** | | **Total households** | **Sampled Households** |  | **Sublocation** | | **Total households** | **Sampled Households** |
| --- | --- | --- | --- | --- | --- | --- | --- | --- |
| 1 | Majengo | 1,258 | 142 |  | 23 | Scheme | 241 | 26 |
| 2 | Wabera | 1,093 | 122 |  | 24 | Gar-Qarsa | 233 | 25 |
| 3 | Rukesa-Qarsa | 895 | 85 |  | 25 | Shura | 308 | 18 |
| 4 | Township | 1,139 | 85 |  | 26 | Gudas/Soriad | 158 | 13 |
| 5 | Bubisa | 1,285 | 72 |  | 27 | Kargi | 876 | 12 |
| 6 | Dakabaricha | 584 | 67 |  | 28 | Leyai | 82 | 10 |
| 7 | Qilta | 541 | 61 |  | 29 | Medate Kuro | 199 | 8 |
| 8 | Qachacha | 528 | 60 |  |  |  |  |  |
| 9 | Nyayo Rd | 490 | 55 |  |  |  |  |  |
| 10 | Dirib Gombo | 452 | 51 |  |  |  |  |  |
| 11 | Karare | 442 | 48 |  |  |  |  |  |
| 12 | Hula-Hula | 417 | 44 |  |  |  |  |  |
| 13 | Logologo | 745 | 41 |  |  |  |  |  |
| 14 | Badasa | 355 | 40 |  |  |  |  |  |
| 15 | Kambinye | 641 | 38 |  |  |  |  |  |
| 16 | Kamboe | 290 | 38 |  |  |  |  |  |
| 17 | Jaldesa | 322 | 37 |  |  |  |  |  |
| 18 | Milima Tatu | 306 | 34 |  |  |  |  |  |
| 19 | Kituruni | 266 | 31 |  |  |  |  |  |
| 20 | Mata_Arba | 272 | 31 |  |  |  |  |  |
| 21 | Songa | 315 | 31 |  |  |  |  |  |
| 22 | Ogicho | 244 | 28 |  |  |  |  |  |

**References**

1. Kenya National Bureau of Statistics (KNBS). The 2009 Kenya Population and Housing Census: VOLUME IC Population Distribution by Age, Sex and Administrative Units. Available at:<http://www.google.com/url?sa=t&rct=j&q=&esrc=s&source=web&cd=2&ved=2ahUKEwizkIOcqYPmAhUEQxUIHaFxCJgQFjABegQIAxAC&url=http%3A%2F%2Fstatistics.knbs.or.ke%2Fnada%2Findex.php%2Fcatalog%2F55%2Fdownload%2F375&usg=AOvVaw3eD4TpqHZR8T5QiAtQSDvr>. Accessed on November 24th, 2019. 2010.

2. World Health Organization (WHO). A Manual for Estimating Disease Burden Associated With Seasonal Influenza. Available at: <https://apps.who.int/iris/bitstream/handle/10665/178801/9789241549301_eng.pdf>. Accessed on Nov, 16th, 2019.

3. Kenya Roads Board (KRB). Map Portal. Available at: ttps://maps.krb.go.ke/kenya-roads-board12769/maps. Accessed on Nov 8, 2022.

4. Kenya Roads Board (KRB). Road Network Classification. Available at: <https://maps.krb.go.ke/kenya-roads-board12769/maps/109276/1-road-network-classification-#>. Accessed on Nov 8, 2022.

5. Lindblade KA, Johnson AJ, Arvelo W, Zhang X, Jordan HT, Reyes L, et al. Low usage of government healthcare facilities for acute respiratory infections in guatemala: implications for influenza surveillance. BMC Public Health. 2011;11:885. Epub 2011/11/25. doi: 10.1186/1471-2458-11-885. PubMed PMID: 22111590; PubMed Central PMCID: PMCPMC3267779.

6. Burton DC, Flannery B, Onyango B, Larson C, Alaii J, Zhang X, et al. Healthcare-seeking behaviour for common infectious disease-related illnesses in rural Kenya: a community-based house-to-house survey. J Health Popul Nutr. 2011;29(1):61-70. PubMed PMID: 21528791; PubMed Central PMCID: PMCPMC3075057.
