# Supplementary material for: Healthcare-seeking behavior for respiratory illnesses in Kenya: implications for burden of disease estimation
Source: BMC Public Health. 2023 Feb 16;23:353. doi: 10.1186/s12889-023-15252-3 (PMC9936639; doi:10.1186/s12889-023-15252-3)
Supplement: Supplementary file 2 — SupplementaryMaterial 2 [file 12889_2023_15252_MOESM2_ESM.docx]

**Supplementary Tables**

**Supplementary Table 1**: Characteristics of the households that participated in the survey by county, Kenya, 2018

| Characteristic | Nakuru (N=1,298) | Marsabit (N=1,353) | Kakamega (N=1,392) | Siaya (N=1,364) | Combined (N=5,407) |
| --- | --- | --- | --- | --- | --- |
|  | n (%) | n (%) | n (%) | n (%) | n (%) |
| Sex of the household head (female) | 277 (21.3) | 327 (24.2) | 544 (39.1) | 749 (54.9) | 1,897 (35.1) |
| Religion |  |  |  |  |  |
| Christian | 1,247 (96.1) | 561 (41.5) | 1,352 (97.1) | 1,357 (99.5) | 4,517 (83.5) |
| Muslim | 21 (1.6) | 714 (52.8) | 39 (2.8) | 6 (0.4) | 780 (14.4) |
| Other | 30 (2.3) | 78 (5.8) | 1 (0.0) | 1 (0.0) | 110 (2.0) |
| Education of the household head |  |  |  |  |  |
| None | 30 (2.3) | 729 (53.9) | 69 (5.0) | 59 (4.3) | 887 (16.4) |
| Pre-primary | 4 (0.3) | 4 (0.3) | 5 (0.4) | 24 (1.8) | 37 (0.7) |
| Primary | 409 (31.5) | 289 (21.4) | 806 (57.9) | 917 (67.2) | 2,421 (44.8) |
| Secondary | 550 (42.4) | 189 (14.0) | 366 (26.3) | 272 (19.9) | 1,377 (25.5) |
| College/University | 270 (20.8) | 137 (10.1) | 140 (10.1) | 83 (6.1) | 630 (11.7) |
| Unknown | 35 (2.7) | 5 (0.4) | 6 (0.4) | 9 (0.7) | 55 (1.0) |
| Main occupation of the household head |  |  |  |  |  |
| Farming/fishing | 112 (8.6) | 66 (4.9) | 458 (32.9) | 517 (37.9) | 1,153 (21.3) |
| Salaried worker | 259 (20.0) | 167 (12.3) | 157 (11.3) | 87 (6.4) | 670 (12.4) |
| Business | 310 (23.9) | 121 (8.9) | 155 (11.1) | 204 (15.0) | 790 (14.6) |
| Skilled/unskilled labor | 547 (42.1) | 512 (37.8) | 426 (30.6) | 317 (23.2) | 1,802 (33.3) |
| Housewife | 21 (1.6) | 153 (11.3) | 88 (6.3) | 97 (7.1) | 359 (6.6) |
| Not Working | 41 (3.2) | 257 (19.0) | 90 (6.5) | 127 (9.3) | 515 (9.5) |
| Other | 8 (0.6) | 77 (5.7) | 18 (1.3) | 15 (1.1) | 118 (2.2) |

**Supplementary Table 2**: Distribution of reported illness syndromes* among survey respondents, by county and age-category, Kenya, 2018

| County | Age (years) | No. of respondents | Had a reported acute respiratory illness in the last 14 days | Had severe pneumonia in the last 12 months |
| --- | --- | --- | --- | --- |
|  |  |  |  |  |
|  |  |  | % (95% CI) | % (95% CI) |
| Combined | <2 | 2,869 | 16.5 (13.8 - 19.7) | 6.8 (5.7 - 8.2) |
|  | 2-4 | 4,409 | 15.9 (13.5 - 18.5) | 8.2 (7.0 - 9.5) |
|  | 5-17 | 8,703 | 7.3 (6.1 - 8.7) | 2.1 (1.8 - 2.6) |
|  | 18-49 | 10,460 | 6.1 (5.2 - 7.3) | 3.3 (2.9 - 3.7) |
|  | ≥50 | 1,631 | 7.6 (6.1 - 9.5) | 5.6 (4.4 - 7.0) |
|  | **All ages** | **28,072** | **9.2 (7.9 - 10.7)** | **4.2 (3.8 - 4.6)** |
| Nakuru | <2 | 742 | 20.8 (14.4 - 29.1) | 9.7 (7.8 - 12.1) |
|  | 2-4 | 906 | 21.0 (15.6 - 27.6) | 12.3 (9.4 - 16.0) |
|  | 5-17 | 1,599 | 9.2 (5.9 - 14.0) | 1.8 (1.1 - 2.8) |
|  | 18-49 | 2,545 | 9.3 (7.1 - 12.0) | 2.8 (2.2 - 3.7) |
|  | ≥50 | 335 | 12.1 (8.1 - 17.6) | 5.4 (3.3 - 8.8) |
|  | **All ages** | **6,127** | **12.6 (9.2 - 16.9)** | **4.9 (4.2 - 5.8)** |
| Marsabit | <2 | 684 | 9.0 (5.2 - 14.9) | 9.0 (6.4 - 12.4) |
|  | 2-4 | 1,226 | 12.3 (8.0 - 18.5) | 9.2 (6.4 - 13.2) |
|  | 5-17 | 2,207 | 5.4 (3.3 - 8.7) | 2.4 (1.7 - 3.3) |
|  | 18-49 | 2,532 | 3.3 (2.2 - 4.9) | 4.3 (3.6 - 5.2) |
|  | ≥50 | 350 | 2.0 (0.9 - 4.5) | 6.0 (3.3 - 10.7) |
|  | **All ages** | **6,999** | **6.0 (3.9 - 9.1)** | **5.1 (4.1 - 6.3)** |
| Kakamega | <2 | 750 | 23.7 (20.5 - 27.3) | 4.8 (3.4 - 6.7) |
|  | 2-4 | 1,119 | 21.3 (18.2 - 24.8) | 5.6 (4.4 - 7.2) |
|  | 5-17 | 2,317 | 10.4 (8.7 - 12.4) | 3.1 (2.2 - 4.2) |
|  | 18-49 | 2,777 | 8.0 (6.8 - 9.3) | 3.0 (2.2 - 4.0) |
|  | ≥50 | 505 | 10.5 (7.8 - 14.1) | 5.9 (3.8 - 9.1) |
|  | **All ages** | **7,468** | **12.5 (11.2 - 13.9)** | **3.8 (3.1 - 4.7)** |
| Siaya | <2 | 693 | 11.7 (9.1 - 14.9) | 3.9 (2.4 - 6.2) |
|  | 2-4 | 1,158 | 10.5 (8.0 - 13.7) | 6.4 (5.0 - 8.2) |
|  | 5-17 | 2,580 | 5.0 (3.9 - 6.4) | 1.3 (0.9 - 1.9) |
|  | 18-49 | 2,606 | 4.0 (3.2 - 5.1) | 3.1 (2.4 - 3.9) |
|  | ≥50 | 441 | 5.7 (3.8 - 8.3) | 5.0 (3.4 - 7.3) |
|  | **All ages** | **7,478** | **6.2 (5.0 - 7.6)** | **3.2 (2.7 - 3.7)** |

*Definitions of illness syndromes in the Methods section

**Supplementary Table 3:** Comparison of reported episodes of respiratory illness, by county, Kenya, 2018

| Age group | County | Number |  | Reported acute respiratory illness in the last 14 days | | |  |  | Reported severe pneumonia in the 12 months | | |
| --- | --- | --- | --- | --- | --- | --- | --- | --- | --- | --- | --- |
|  |  |  | % | | Odds Ratio (95% CI) | p-value |  | % | | Odds Ratio (95% CI) | p-value |
| <5 years | Nakuru | 1,664 | 21.3 (15.9 – 28.1) | | Ref |  |  | 11.2 (9.1 – 13.7) | | Ref | Ref |
|  | Marsabit | 1,918 | 11.3 (7.4 – 16.8) | | 0.47 (0.26 - 0.85) | 0.017 |  | 9.1 (6.9 – 12.0) | | 0.80 (0.55 - 1.16) | 0.442 |
|  | Kakamega | 1,872 | 22.4 (20.0 – 24.9) | | 1.06 (0.72 - 1.57) | 0.758 |  | 5.2 (4.1 – 6.5) | | 0.43 (0.31 - 0.60) | <0.001 |
|  | Siaya | 1,862 | 11.1 (9.0 – 13.5) | | 0.46 (0.30 - 0.71) | <0.001 |  | 5.6 (4.3 – 7.1) | | 0.47 (0.33 - 0.66) | <0.001 |
| 5-49 years | Nakuru | 4,159 | 9.4 (6.9 – 12.6) | | Ref |  |  | 2.1 (1.6 – 3.0) | | Ref | Ref |
|  | Marsabit | 4,751 | 4.3 (2.8 – 6.4) | | 0.43 (0.25 - 0.75) | 0.004 |  | 2.7 (2.2 – 3.4) | | 1.29 (0.88 - 1.89) | 0.075 |
|  | Kakamega | 5,105 | 9.2 (8.0 – 10.6) | | 0.98 (0.68 - 1.42) | 0.926 |  | 2.9 (2.2 – 3.8) | | 1.36 (0.89 - 2.07) | 0.150 |
|  | Siaya | 5,203 | 4.6 (3.7 – 5.6) | | 0.46 (0.31 - 0.69) | <0.001 |  | 1.9 (1.6 – 2.4) | | 0.88 (0.60 - 1.29) | 0.512 |
| ≥50 years | Nakuru | 335 | 12.1 (8.1 – 17.6) | | Ref |  |  | 5.4 (3.3 – 8.8) | | Ref | Ref |
|  | Marsabit | 352 | 2.0 (0.9 – 4.5) | | 0.17 (0.07 - 0.43) | <0.001 |  | 6.0 (3.3 – 10.7) | | 1.10 (0.46 -2.60) | 0.875 |
|  | Kakamega | 507 | 10.5 (7.8 – 14.1) | | 0.85 (0.50 - 1.46) | 0.555 |  | 5.9 (3.8 – 9.1) | | 1.26 (0.59 -2.71) | 0.548 |
|  | Siaya | 446 | 5.7 (3.8 – 8.3) | | 0.47 (0.25 - 0.87) | 0.016 |  | 5.0 (3.4 – 7.3) | | 1.13 (0.56 -2.29) | 0.735 |
| All ages | Nakuru | 6,158 | 12.6 (8.1 – 16.9) | | Ref | Ref |  | 4.9 (4.2 – 5.8) | | Ref | Ref |
|  | Marsabit | 7,021 | 6.0 (3.9 – 9.1) | | 0.44 (0.26 - 0.76) | 0.005 |  | 5.1 (4.1 – 6.3) | | 1.07 (0.82 -1.30) | 0.686 |
|  | Kakamega | 7,484 | 12.5 (11.2 – 13.9) | | 0.98 (0.69 - 1.40) | 0.930 |  | 3.8 (3.1 – 4.7) | | 0.77 (0.57 -1.03) | 0.082 |
|  | Siaya | 7,511 | 6.2 (5.0 – 7.6) | | 0.46 (0.31 - 0.67) | <0.001 |  | 3.2 (2.7 – 3.7) | | 0.62 (0.48 -0.81) | <0.001 |

**Supplementary Table 4:** Comparison of healthcare seeking for respiratory illness, by county, Kenya, 2018

|  |  |  |  | Acute respiratory illness in last 14 days | | |  |  | | Severe pneumonia in the last 12 months | | | |
| --- | --- | --- | --- | --- | --- | --- | --- | --- | --- | --- | --- | --- | --- |
| Age group | County | Number |  | Sought healthcare at a health facility | | |  | Number |  | | Sought healthcare at a health facility | | |
|  |  |  | % (95% CI) | | Odds Ratio (95% CI) | p-value |  |  | % (95% CI) | | | Odds Ratio (95% CI) | p-value |
| <5 years | Nakuru | 354 | 52.5 (44.2 – 60.6) | | Ref |  |  | 182 | 87.6 (80.7 – 92.3) | | | Ref | Ref |
|  | Marsabit | 216 | 52.7 (46.0 – 59.5) | | 1.01 (0.66 - 1.55) | 0.684 |  | 174 | 82.1 (76.1 – 87.0) | | | 0.65 (0.34 - 1.24) | 0.276 |
|  | Kakamega | 419 | 34.4 (29.4 – 39.7) | | 0.47 (0.32 - 0.71) | <0.001 |  | 99 | 76.3 (66.0 – 84.1) | | | 0.45 (0.22 - 0.94) | 0.034 |
|  | Siaya | 206 | 50.5 (40.4 – 60.5) | | 0.92 (0.55 - 1.56) | 0.763 |  | 100 | 67.0 (55.7 – 76.6) | | | 0.29 (0.14 - 0.58) | 0.001 |
| 5-49 years | Nakuru | 381 | 35.6 (30.7 – 40.1) | | Ref |  |  | 88 | 75.6 (61.0 – 86.0) | | | Ref | Ref |
|  | Marsabit | 203 | 55.7 (46.8 – 64.5) | | 2.27 (1.48 - 3.50) | <0.001 |  | 133 | 81.1 (72.4 – 87.5) | | | 1.38 (0.60 - 3.21) | 0.633 |
|  | Kakamega | 471 | 26.1 (21.1 – 31.8) | | 0.64 (0.45 - 0.91) | 0.014 |  | 147 | 51.3 (40.9 – 61.6) | | | 0.34 (0.15 - 0.76) | 0.006 |
|  | Siaya | 237 | 35.0 (27.5 – 43.3) | | 0.98 (0.64 - 1.48) | 0.907 |  | 100 | 62 (51.7 – 71.3) | | | 0.53 (0.24 - 1.18) | 0.087 |
| ≥50 years | Nakuru | 41 | 42.1 (27.1 – 58.7) | | Ref |  |  | 16 | 76.5 (48.0 - 92.0) | | | Ref | Ref |
|  | Marsabit | 8 | 62.5 (29.7 – 86.9) | | 2.29 (0.50 - 10.56) | 0.285 |  | 17 | 81.0 (66.0 - 90.3) | | | 1.27 (0.27 - 6.00) | 0.759 |
|  | Kakamega | 53 | 22.6 (13.9 – 34.6) | | 0.40 (0.16 - 0.99) | 0.046 |  | 28 | 66.7 (47.4 - 81.6) | | | 0.58 (0.13 - 2.61) | 0.472 |
|  | Siaya | 27 | 40.7 (19.6 – 65.9) | | 0.95 (0.28 - 3.25) | 0.928 |  | 22 | 54.5 (37.5 - 70.6) | | | 0.33 (0.08 - 1.38) | 0.127 |
| All ages | Nakuru | 776 | 43.6 (37.9 – 49.5) | | Ref |  |  | 286 | 84.2 (76.4 - 89.8) | | | Ref | Ref |
|  | Marsabit | 427 | 54.3 (47.9 – 60.7) | | 1.54 (1.08 - 2.19) | 0.004 |  | 324 | 82.1 (76.7 - 86.7) | | | 0.89 (0.50 - 1.58) | 0.629 |
|  | Kakamega | 943 | 29.6 (25.4 – 34.1) | | 0.54 (0.40 - 0.75) | <0.001 |  | 274 | 63.0 (53.6 - 71.6) | | | 0.32 (0.17 - 0.60) | <0.001 |
|  | Siaya | 470 | 42.1 (34.5 – 50.1) | | 0.94 (0.63 - 1.40) | 0.767 |  | 222 | 64.4 (58.1 - 70.2) | | | 0.35 (0.20 - 0.61) | <0.001 |

**Supplementary Table 5:** Factors associated with healthcare-seeking behavior for acute respiratory illness, by county, Kenya, 2018

| County | Characteristic | | ^a^Number | Went to any health facility (public or private) | | | Went to a private health facility | | | Purchased drugs from a pharmacy/shop | | |  |
| --- | --- | --- | --- | --- | --- | --- | --- | --- | --- | --- | --- | --- | --- |
|  |  |  |  |  |  |  |  |  |  |  |  |  |  |
|  |  |  |  | % | Adjusted Odds Ratio* (95% CI) | p-value | % | Adjusted Odds Ratio* (95% CI) | p-value | % | Adjusted Odds Ratio* (95% CI) | p-value |  |
| Nakuru | Sex | Male | 352 | 47.5 | Ref |  | 14.7 | Ref |  | 16.7 | Ref |  |  |
|  |  | Female | 423 | 40.4 | 0.81 (0.59 - 1.10) | 0.174 | 11.3 | 0.84 (0.58 - 1.22) | 0.361 | 15.7 | 0.90 (0.67 - 1.20) | 0.453 |  |
|  | Age (years) | <5 years | 354 | 52.5 | 1.96 (1.45 - 2.66) | <0.001 | 14.7 | 1.31 (0.71 - 2.41) | 0.379 | 13.8 | 0.69 (0.45 - 1.07) | 0.098 |  |
|  |  | 5-49 years | 380 | 35.7 | Ref |  | 11.1 | Ref |  | 18.4 | Ref |  |  |
|  |  | ≥50 years | 41 | 42.1 | 1.27 (0.66 - 2.47) | 0.463 | 13.2 | 1.40 (0.54 - 3.59) | 0.475 | 15.8 | 0.85 (0.36 - 2.00) | 0.697 |  |
|  | ^b^Education level | Low | 340 | 47.6 | Ref |  | 7.1 | Ref |  | 15.0 | Ref |  |  |
|  |  | High | 435 | 40.7 | 0.73 (0.48 - 1.10) | 0.127 | 17.3 | 2.05 (1.24 - 3.37) | 0.006 | 17.0 | 1.09 (0.81 - 1.48) | 0.565 |  |
|  | ^c^Household SES | Low | 529 | 44.2 | Ref |  | 7.9 | Ref |  | 14.9 | Ref |  |  |
|  |  | High | 246 | 42.5 | 0.99 (0.71 - 1.37) | 0.936 | 23.3 | 2.95 (1.82 - 4.78) | <0.001 | 18.8 | 1.29 (0.73 - 2.28) | 0.379 |  |
| Marsabit | Sex | Male | 224 | 51.7 | Ref |  | 9.8 | Ref |  | 13.6 | Ref |  |  |
|  |  | Female | 203 | 61.4 | 1.50 (1.02 - 2.22) | 0.041 | 12.1 | 1.16 (0.50 - 2.70) | 0.721 | 10.7 | 0.60 (0.32 - 1.14) | 0.114 |  |
|  | Age (years) | <5 years | 216 | 54.8 | 0.93 (0.62 - 1.39) | 0.715 | 8.7 | 0.59 (0.35 - 1.00) | 0.048 | 15.2 | 1.50 (0.81 - 2.79) | 0.191 |  |
|  |  | 5-49 years | 203 | 57.8 | Ref |  | 13.2 | Ref |  | 8.0 | Ref |  |  |
|  |  | ≥50 years | 8 | 62.5 | 1.27 (0.31 - 5.20) | 0.727 | 12.5 | 1.02 (0.11 - 9.70) | 0.984 | 37.5 | 10.78 (1.76 - 66.21) | 0.012 |  |
|  | ^b^Education level | Low | 339 | 56.7 | Ref |  | 10.3 | Ref |  | 9.2 | Ref |  |  |
|  |  | High | 88 | 54.8 | 0.96 (0.45 - 2.05) | 0.913 | 12.9 | 1.16 (0.39 - 3.49) | 0.780 | 23.7 | 1.58 (0.80 - 3.12) | 0.176 |  |
|  | ^c^Household SES | Low | 273 | 56.9 | Ref |  | 9.7 | Ref |  | 5.9 | Ref |  |  |
|  |  | High | 154 | 55.3 | 0.91 (0.59 - 1.40) | 0.654 | 13.0 | 1.43 (0.42 - 4.88) | 0.551 | 23.6 | 4.79 (2.05 - 11.20) | 0.001 |  |
| Kakamega | Sex | Male | 437 | 29.1 | Ref |  | 5.7 | Ref |  | 29.8 | Ref |  |  |
|  |  | Female | 506 | 30.0 | 1.08 (0.77 - 1.52) | 0.649 | 3.8 | 0.69 (0.32 - 1.48) | 0.329 | 25.9 | 0.83 (0.60 - 1.17) | 0.283 |  |
|  | Age (years) | <5 years | 419 | 34.4 | 1.45 (1.10 - 1.93) | 0.011 | 5.5 | 1.37 (0.79 - 2.35) | 0.254 | 26.5 | 0.88 (0.68 - 1.15) | 0.345 |  |
|  |  | 5-49 years | 471 | 26.1 | Ref |  | 3.8 | Ref |  | 28.2 | Ref |  |  |
|  |  | ≥50 years | 53 | 22.6 | 0.83 (0.47 - 1.48) | 0.520 | 5.7 | 1.47 (0.38 - 5.63) | 0.568 | 32.1 | 1.06 (0.55 - 2.05) | 0.863 |  |
|  | ^b^Education level | Low | 614 | 26.9 | Ref |  | 3.4 | Ref |  | 27.9 | Ref |  |  |
|  |  | High | 329 | 34.7 | 1.35 (0.97 - 1.86) | 0.070 | 7.0 | 1.75 (0.85 - 3.60) | 0.124 | 27.4 | 0.83 (0.59 - 1.18) | 0.305 |  |
|  | ^c^Household SES | Low | 566 | 27.4 | Ref |  | 3.2 | Ref |  | 23.9 | Ref |  |  |
|  |  | High | 377 | 32.9 | 1.19 (0.83 - 1.70) | 0.334 | 6.9 | 1.82 (0.92 - 3.62) | 0.085 | 33.4 | 1.68 (1.18 - 2.39) | 0.005 |  |
| Siaya | Sex | Male | 217 | 37.3 | Ref |  | 0.9 | Ref |  | 26.7 | Ref |  |  |
|  |  | Female | 252 | 46.4 | 1.50 (1.04 - 2.17) | 0.031 | 2.0 | 2.20 (0.31 - 15.53) | 0.419 | 25.8 | 0.94 (0.65 - 1.36) | 0.741 |  |
|  | Age (years) | <5 years | 205 | 50.7 | 2.04 (1.40 - 2.97) | <0.001 | 1.5 | 0.75 (0.14 - 4.04) | 0.733 | 18.5 | 0.47 (0.29 - 0.76) | 0.003 |  |
|  |  | 5-49 years | 237 | 35.0 | Ref |  | 1.7 | Ref |  | 32.1 | Ref |  |  |
|  |  | ≥50 years | 27 | 40.7 | 1.24 (0.43 - 3.54) | 0.687 | 0.0 | NE | - | 33.3 | 0.90 (0.38 - 2.12) | 0.800 |  |
|  | ^b^Education level | Low | 363 | 42.4 | Ref |  | 1.1 | Ref |  | 28.1 | Ref |  |  |
|  |  | High | 106 | 41.5 | 0.88 (0.57 - 1.35) | 0.545 | 2.8 | 2.54 (0.81 - 7.96) | 0.106 | 19.8 | 0.64 (0.35 - 1.15) | 0.131 |  |
|  | ^c^Household SES | Low | 277 | 44.8 | Ref |  | 1.4 | Ref |  | 22.0 | Ref |  |  |
|  |  | High | 192 | 38.5 | 0.72 (0.41 - 1.26) | 0.241 | 1.6 | 0.94 (0.18 - 4.96) | 0.937 | 32.3 | 1.95 (1.03 - 3.68) | 0.041 |  |

^a^Number who reported an episode of acute respiratory illness in the 14 days preceding the survey; ^b^Level of education for the head of the household (Low=Primary level or none, High=Secondary level or higher); ^c^Household socio-economic status (Low= 1-3 quintile, High=4-5 quintile); *Adjusted for all variables listed; NE – Not estimated

**Supplementary Table 6:** Factors associated with healthcare-seeking behavior for severe pneumonia in the last 12 months, by county, Kenya, 2018

| County | Characteristic |  | ^a^Number | Went to any health facility (public of private) | | | Went to a private health facility | | | Hospitalized | | |
| --- | --- | --- | --- | --- | --- | --- | --- | --- | --- | --- | --- | --- |
|  |  |  |  | % | Adjusted Odds Ratio* (95% CI) | p-value | % | Adjusted Odds Ratio* (95% CI) | p-value | % | Adjusted Odds Ratio* (95% CI) | p-value |
| Nakuru | Sex | Male | 147 | 85.5 | Ref |  | 41.3 | Ref |  | 24.8 | Ref |  |
|  |  | Female | 150 | 81.9 | 0.85 (0.38 - 1.89) | 0.681 | 39.9 | 0.90 (0.50 - 1.62) | 0.851 | 17.2 | 0.72 (0.34 - 1.29) | 0.140 |
|  | Age (years) | <5 years | 100 | 76.6 | 2.01 (1.08 - 3.72) | 0.028 | 45.5 | 1.65 (1.02 - 2.68) | 0.038 | 23.6 | 1.29 (0.73 - 2.29) | 0.358 |
|  |  | 5-49 years | 178 | 87.6 | Ref |  | 33.7 | Ref |  | 17.4 | Ref |  |
|  |  | ≥50 years | 19 | 78.6 | 1.16 (0.29 - 4.71) | 0.826 | 21.4 | 0.56 (0.11 - 2.83) | 0.472 | 14.3 | 0.77 (0.15 - 3.88) | 0.757 |
|  | ^b^Education level | Low | 116 | 78.1 | Ref |  | 25.7 | Ref |  | 14.3 | Ref |  |
|  |  | High | 181 | 87.3 | 1.79 (0.99 - 3.23) | 0.053 | 49.7 | 1.87 (1.04 - 3.34) | 0.036 | 25.4 | 1.86 (1.07 - 3.24) | 0.024 |
|  | ^c^Household SES | Low | 186 | 82.1 | Ref |  | 26.5 | Ref |  | 19.2 | Ref |  |
|  |  | High | 111 | 85.8 | 1.06 (0.52 - 2.17) | 0.860 | 57.5 | 3.18 (1.68 - 6.05) | 0.001 | 23.6 | 1.04 (0.53 - 2.04) | 0.849 |
| Marsabit | Sex | Male | 177 | 82.5 | Ref |  | 11.7 | Ref |  | 29.8 | Ref |  |
|  |  | Female | 173 | 81.1 | 0.93 (0.56 - 1.53) | 0.762 | 20.9 | 2.12 (1.35 - 3.34) | 0.009 | 18.3 | 0.49 (0.28 - 0.84) | 0.012 |
|  | Age (years) | <5 years | 168 | 82.2 | 1.06 (0.56 - 2.00) | 0.854 | 16.8 | 1.26 (0.52 - 3.06) | 0.601 | 25.3 | 1.20 (0.66 - 2.16) | 0.537 |
|  |  | 5-49 years | 161 | 81.2 | Ref |  | 15.1 | Ref |  | 20.3 | Ref |  |
|  |  | ≥50 years | 21 | 82.4 | 1.11 (0.37 - 3.34) | 0.849 | 17.7 | 1.14 (0.43 - 3.01) | 0.782 | 47.1 | 3.95 (1.63 - 9.55) | 0.004 |
|  | ^b^Education level | Low | 271 | 82.0 | Ref |  | 17.6 | Ref |  | 23.1 | Ref | 0.780 |
|  |  | High | 79 | 81.2 | 1.11 (0.48 - 2.55) | 0.797 | 10.1 | 0.74 (0.34 - 1.59) | 0.427 | 29.0 | 1.09 (0.59 - 2.02) |  |
|  | ^c^Household SES | Low | 206 | 84.1 | Ref |  | 21.0 | Ref |  | 19.8 | Ref |  |
|  |  | High | 144 | 79.6 | 0.72 (0.34 - 1.49) | 0.361 | 11.4 | 0.51 (0.22 - 1.19) | 0.113 | 28.7 | 1.66 (1.06 - 2.58) | 0.027 |
| Kakamega | Sex | Male | 137 | 60.7 | Ref |  | 20.7 | Ref |  | 20.7 | Ref |  |
|  |  | Female | 140 | 62.8 | 1.38 (0.87 - 2.21) | 0.167 | 20.0 | 1.16 (0.52 - 2.61) | 0.709 | 19.3 | 1.24 (0.66 - 2.33) | 0.505 |
|  | Age (years) | <5 years | 93 | 76.3 | 3.23 (2.08 - 5.02) | <0.001 | 30.9 | 3.52 (1.76 - 7.05) | 0.001 | 34.0 | 3.89 (2.03 - 7.45) | <0.001 |
|  |  | 5-49 years | 154 | 51.3 | Ref |  | 11.3 | Ref |  | 12.7 | Ref |  |
|  |  | ≥50 years | 30 | 67.9 | 1.90 (0.84 - 4.29) | 0.121 | 32.1 | 3.64 (1.32 - 10.05) | 0.013 | 10.7 | 0 .83 (0.23 - 3.01) | 0.772 |
|  | ^b^Education level | Low | 177 | 64.4 | Ref |  | 19.0 | Ref |  | 21.8 | Ref |  |
|  |  | High | 100 | 57.4 | 0.63 (0.38 - 1.05) | 0.076 | 22.8 | 1.17 (0.52 - 2.63) | 0.700 | 16.8 | 0.70 (0.31 - 1.61) | 0.399 |
|  | ^c^Household SES | Low | 161 | 59.0 | Ref |  | 17.3 | Ref |  | 20.9 | Ref |  |
|  |  | High | 116 | 64.7 | 1.31 (0.74 - 2.33) | 0.346 | 23.5 | 1.18 (0.57 - 2.44) | 0.646 | 19.1 | 0.85 (0.42 - 1.74) | 0.657 |
| Siaya | Sex | Male | 100 | 63.8 | Ref |  | 18.1 | Ref |  | 23.4 | Ref |  |
|  |  | Female | 135 | 63.4 | 0.91 (0.57 - 1.48) | 0.709 | 16.0 | 0.67 (0.26 - 1.71) | 0.390 | 19.1 | 0.65 (0.33 - 1.29) | 0.214 |
|  | Age (years) | <5 years | 99 | 67.0 | 1.36 (0.65 - 2.83) | 0.408 | 16.5 | 1.10 (0.46 - 2.67) | 0.821 | 22.3 | 1.45 (0.55 - 3.81) | 0.438 |
|  |  | 5-49 years | 114 | 62.0 | Ref |  | 16.0 | Ref |  | 17.0 | Ref |  |
|  |  | ≥50 years | 22 | 54.6 | 0.75 (0.35 - 1.62) | 0.452 | 22.7 | 1.65 (0.50 - 5.42) | 0.402 | 31.8 | 2.54 (0.80 - 8.03) | 0.111 |
|  | ^b^Education level | Low | 174 | 60.5 | Ref |  | 12.6 | Ref |  | 19.2 | Ref |  |
|  |  | High | 61 | 72.4 | 1.98 (1.06 - 3.70) | 0.032 | 29.3 | 2.96 (1.16 - 7.54) | 0.024 | 25.9 | 1.57 (0.78 - 3.16) | 0.196 |
|  | ^c^Household SES | Low | 139 | 65.5 | Ref |  | 13.8 | Ref |  | 18.9 | Ref |  |
|  |  | High | 96 | 61.2 | 0.71 (0.38 - 1.34) | 0.283 | 20.2 | 1.25 (0.55 - 2.85) | 0.583 | 20.9 | 1.11 (0.41 - 2.98) | 0.839 |

^a^Number who reported an episode of acute respiratory illness in the 14 days preceding the survey; ^b^Level of education for the head of the household (Low=Primary level or none, High=Secondary level or higher); ^c^Household socio-economic status (Low= 1-3 quintile, High=4-5 quintile); *Adjusted for all variables listed

**Supplementary Table 7**: Other factors explored for possible association with healthcare-seeking behavior for acute respiratory illness^a^, Kenya, 2018

| Characteristic |  | Adjusted Odds Ratio* (95% CI) | p-value | Adjusted Odds Ratio* (95% CI) | p-value | Adjusted Odds Ratio* (95% CI) | p-value |
| --- | --- | --- | --- | --- | --- | --- | --- |
| Religion | Christian | Ref |  | Ref |  | Ref |  |
|  | Muslim | 0.81 (0.49 - 1.34) | 0.402 | 0.80 (0.44 - 1.46) | 0.472 | 1.24 (0.70 - 2.20) | 0.459 |
|  | Other | NE |  | NE |  | NE |  |
| Household size | Small | Ref |  | Ref |  | Ref |  |
|  | Large^b^ | 0.97 (0.79 - 1.18) | 0.735 | 1.00 (0.70 - 1.44) | 0.986 | 1.04 (0.83 - 1.31) | 0.728 |
| Household membership | Other relative | Ref |  | Ref |  | Ref |  |
|  | Immediate family member | 0.81 (0.64 - 1.03) | 0.079 | 0.67 (0.42 - 1.06) | 0.084 | 0.70 (0.26 - 1.92) | 0.491 |
| Child birth order^c^ | Order number | 1.02 (0.95 - 1.11) | 0.581 | 0.93 (0.81 - 1.07) | 0.319 | 0.96 (0.89 - 1.05) | 0.380 |
| Distance to the nearest road (meters)^c^ | Meters | 1.00 (1.00 - 1.00) | 0.364 | 1.00 (1.00 - 1.00) | 0.050 | 1.00 (1.00 - 1.00) | 0.246 |

^a^Combines data for all the four sites (counties); ^b^More than median number of household members; ^c^Modeled as a continuous variable; *Adjusted for site (county); NE – Not estimated

**Supplementary Table 8:** Other factors explored for possible association with healthcare-seeking behavior for severe pneumonia in the last 12 months^a^, Kenya, 2018

| Characteristic |  | Adjusted Odds Ratio* (95% CI) | p-value | *Adjusted Odds Ratio* (95% CI) | p-value | *Adjusted Odds Ratio* (95% CI) | p-value |
| --- | --- | --- | --- | --- | --- | --- | --- |
| Religion | Christian | Ref |  | Ref |  | Ref |  |
|  | Muslim | 0.70 (0.38 - 1.28) | 0.240 | 0.60 (0.31 - 1.17) | 0.136 | 1.11 (0.75 - 1.65) | 0.592 |
|  | Other | NE |  | NE |  | NE |  |
| Household size | Small | Ref |  | Ref |  | Ref |  |
|  | Large^b^ | 0.97 (0.72 - 1.32) | 0.861 | 0.73 (0.51 - 1.04) | 0.081 | 0.95 (0.68 - 1.34) | 0.784 |
| Household membership | Other relative | Ref |  | Ref |  | Ref |  |
|  | Immediate member | 0.80 (0.49 - 1.31) | 0.380 | 1.06 (0.65 - 1.73) | 0.809 | 0.82 (0.54 - 1.24) | 0.339 |
| Child birth order^c^ | Order number | 1.04 (0.90 - 1.19) | 0.604 | 0.96 (0.85 - 1.09) | 0.566 | 1.01 (0.90 - 1.13) | 0.914 |
| Distance to the nearest road (meters)^c^ | Meters | 1.00 (1.00 - 1.00) | 0.570 | 1.00 (1.00 - 1.00) | 0.588 | 1.00 (1.00 - 1.00) | 0.260 |

^a^Combines data for all the four sites (counties); ^b^More than median number of household members; ^c^Modeled as a continuous variable; *Adjusted for site (county); NE – Not estimated
